# Stomata‐Photosynthesis Synergy Mediates Combined Heat and Salt Stress Tolerance in Sugarcane Mutant M4209

**DOI:** 10.1111/pce.15424

**Published:** 2025-03-07

**Authors:** Pooja Negi, Manish Pandey, Radha K. Paladi, Arnab Majumdar, Shailaja P. Pandey, Vitthal T. Barvkar, Rachayya Devarumath, Ashish K. Srivastava

**Affiliations:** ^1^ Nuclear Agriculture and Biotechnology Division Bhabha Atomic Research Centre Mumbai India; ^2^ Homi Bhabha National Institute Mumbai India; ^3^ School of Environmental Studies Jadavpur University Kolkata India; ^4^ Analytical Chemistry Division Bhabha Atomic Research Centre Mumbai India; ^5^ Department of Botany Savitribai Phule Pune University Pune India; ^6^ Vasantdada Sugar Institute Pune India

**Keywords:** hydraulic synergy, lipid reprogramming, photosynthetic efficiency, source strength, stomatal density, transpirational cooling, vapour pressure deficit

## Abstract

Sugarcane (*Saccharum officinarum* L.) is an economically important long‐duration crop which is currently facing concurrent heat waves and soil salinity. The present study evaluates an inducible salt‐tolerant sugarcane mutant M4209, developed via radiation‐induced mutagenesis of elite check variety Co 86032, under heat (42/30°C; day/night), NaCl (200 mM) or heat + NaCl (HS)‐stress conditions. Though heat application significantly improved plant growth and biomass in both genotypes, this beneficial impact was partially diminished in Co 86032 under HS‐stress conditions, coinciding with higher Na^+^ accumulation and lower triacylglycerol levels. Besides, heat broadly equalised the negative impact on NaCl stress in terms of various physiological and biochemical attributes in both the genotypes, indicating its spaciotemporal advantage. The simultaneous up‐ and downregulation of antagonistic regulators, *epidermal patterning factor (EPF) 9 (SoEPF9)* and *SoEPF2*, respectively attributed to the OSD (Open Small Dense) stomatal phenotype in M4209, which resulted into enhanced conductance, transpirational cooling and gaseous influx. This led to improved photoassimilation, which was supported by higher plastidic:nonplastidic lipid ratio, upregulation of *SoRCA* (Rubisco activase) and better source strength, resulting in overall plant growth enhancement across all the tested stress scenarios. Taken together, the present study emphasised the knowledge‐driven harnessing of stomatal‐photosynthetic synergy for ensuring global sugarcane productivity, especially under “salt‐heat” coupled stress scenarios.

## Summary statement

The study evaluated M4209, a radiation induced salt‐tolerant sugarcane mutant and its salt‐sensitive elite parent Co 86032, under heat‐NaCl stress combination. Heat broadly equalised the negative impact of NaCl stress, with the beneficial impact being partially diminished in Co 86032. The OSD (Open Small Dense) stomatal phenotype in M4209 supported enhanced transpirational cooling, gaseous influx and photoassimilation, boosting plant growth across the tested stress scenarios.

## 1 Introduction

Global food safety is at‐risk from the rising population amidst climate perturbations, including soil salinity and warming temperatures (IPCC 2021; Zandalinas et al. 2021). The latter is projected to increase by approximately 0.3°C–4.8°C by 2100 (Jagadish et al. 2021), with progressively frequent occurrences of intense and/or prolonged heatwaves. Thus, crops will likely be simultaneously or sequentially exposed to high temperatures with soil salinity (Zandalinas et al. 2021; Zandalinas and Mittler 2022), with serious implications for sustainable agricultural production (Hopmans et al. 2021). The individual effects of heat and salt stress has been well‐investigated in plants. Heat‐stress affects chlorophyll biosynthesis, thylakoid membrane stability, photochemical reactions, and Calvin cycle metabolism (Qu et al. 2021, Zahra et al. 2023). The impaired function of Rubisco (ribulose‐1,5‐biphosphate carboxylase/oxygenase) and its poor activation by Rubisco activase was identified as one of the major causes of heat‐induced photosynthetic limitation (Qu et al. 2021, Zahra et al. 2023). Similarly, soil salinity limits photosynthesis in several ways, including oxidative damage to photosynthetic apparatus, reduced chlorophyll biosynthesis, impaired photosystem functionality, restricted electron transport, and non‐photochemical quenching (Van Zelm et al. 2020). On the other hand, stomatal dynamics also impact photosynthetic efficiency, leaf thermoregulation and intrinsic water‐use efficiency (iWUE), thereby regulating plant responses to these stress conditions (Faralli et al. 2019; Wang et al. 2022; Caine et al. 2023).

Till date, limited studies have investigated crop responses to combined heat and NaCl (HS)‐stress conditions, and recent reports hint at both antagonistic and synergistic interactions (Zandalinas et al. 2021; Rivero et al. 2022; Zandalinas and Mittler 2022; Pardo‐Hernández et al. 2024). For instance, HS‐stress conditions have been shown to reduce K^+^/Na^+^ and chlorophyll content (Suzuki et al. 2016), severely limiting plant growth and yield in *Arabidopsis* (Esmaeili et al. 2019; Wijewardene et al. 2020). Heat stress aggravated NaCl‐related toxicity by increasing transpiration‐associated Cl^−^ intake in citrange (Balfagón et al. 2019) and inducing ionic and redox imbalance in rice (Jan et al. 2021). In contrast, HS‐stressed tomato plants showed improved growth over salt‐stressed plants, which was attributed to better osmotic adjustment and higher K^+^/Na^+^ ratio (Rivero et al. 2014). In rice, the protection of photosystems at the cost of higher transpiration rate led to improved growth and biomass under HS‐stress conditions (Nahar et al. 2022). These studies highlighted photosynthetic efficiency and ionic homoeostasis as major factors driving interactions between heat and NaCl stress conditions.

Both heat and NaCl stress are known to modulate the developmental plasticity and aperture kinetics of stomata, which fine‐tunes the physiological trade‐off between gas influx and transpirational cooling under these stress conditions (Faralli et al. 2019). Salt stress is generally hypothesised to reduce stomatal density and force stomatal closure to prevent water loss and Na^+^ accumulation in leaves, though it is species‐ and context‐specific (Hasanuzzaman et al. 2023). By contrast, heat is thought to increase stomatal conductance, which promotes transpirational cooling, enabling thermoregulation and photosynthesis, but in a VPD (vapour pressure deficit)‐dependent and species‐specific manner (Urban et al. 2017; Caine et al. 2023). In *Arabidopsis*, the increased triacylglycerol (TG) accumulation and metabolism was shown to be essential for stomatal dynamics at elevated temperatures (Korte et al. 2023). The maintenance of stomatal density and/or faster stomatal movement, resulting in better stomatal conductance was linked to improved salt tolerance (Kiani‐Pouya et al. 2020). Several genetically‐modified plants with improved heat and/or NaCl stress tolerance have been shown to have altered stomatal traits. For instance. rice *hst1* (heat stress‐tolerant 1) mutant showed improved heat tolerance due to improved redox balance, water homoeostasis and stomatal closure (Ding et al. 2023). In rice, overexpression of histone deacetylase *HDA704* improved both drought and salt tolerance (Zhao et al. 2021), while the overexpression of *AtICE1* enhanced yield and multi‐stress tolerance (Verma et al. 2020), via controlling stomatal aperture and density. In the same line, suppression of stomatal density, either by overexpression of suppressor peptides (*EPF1*/*EP2)* or their upstream regulators (*MYC2*) was shown to improve water use efficiency and drought tolerance in several crops (Caine et al. 2023; Ferguson et al. 2024; Xia et al. 2024), indicating the complex regulation of stomatal traits under heat and/or salt stress conditions.

**Figure 1:**
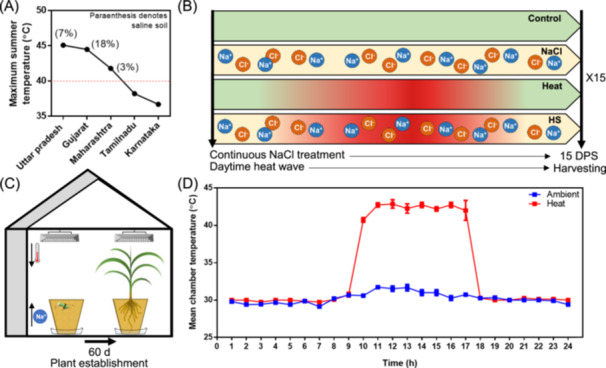
Theoretical rationale and experimental layout for individual and combined NaCl and heat stress regime. The average maximum summer temperature of major sugarcane cultivating states in India. Parenthesis indicates the percent of total saline soils in India, accounted for by a particular state (A). Schematic representation of single and combined stress regimes. At the six‐leaf tillering stage (Approx. 60 d post‐germination), uniformly‐grown plants were organised into four independent sets and subjected to respective stress regime, followed by plant phenotyping and harvesting at 15 d post‐stress (B). Schematic representation of the experimental setup used for stress treatments. Control plants were irrigated with half‐strength Murashige and Skoog medium and maintained at approximately 30°C throughout the experiment. NaCl stress was imposed by incrementally saturating soil with NaCl solution present in below‐pot reservoirs, with final concentration of 200 mM NaCl. To simulate day‐time heat waves, the chamber temperature was daily raised from 30°C to 42°C at 9 AM in the morning using overhead infrared lamps (ramp rate 0.2°C min^−1^), and returned to 30°C at 5 PM in the evening (heat). Arrowhead indicates the directionality of stress: heat stress: shootborne; NaCl stress: rootborne. (C) Graph representing the average hourly temperatures of the green house chamber during the study (D). Error bar indicates standard error representative of day‐wise fluctuation in chamber temperature (*N* = 15).

Sugarcane (*Saccharum officinarum* L.) is a cash crop of global socio‐economic relevance, which is cultivated over approximately 26 million hectares of arable land spread across 120 countries (UN Food and Agriculture Organization, Corporate Statistical Database [FAOSTAT] 2022); Indian sugarcane accounts for approx. 19.76% of world production (Directorate of Economics & Statistics, Government of India 2020). Though, sugarcane responses to soil salinity are well‐characterised, the impact of heat stress in sugarcane and corresponding thermo‐adaptive responses are relatively less explored. Additionally, major sugarcane cultivating areas are experiencing the co‐occurrence of elevated temperatures and soil salinity (Figure 1A; Chakraborty et al. 2019; Kumar and Sharma 2020). However, the effect of HS‐stress on sugarcane and corresponding adaptive strategies have not been investigated in detail. To address these lacunae, we performed a comparative post‐germination stress profiling of the elite but salt‐sensitive cultivar, Co 86032 and its salt‐tolerant mutant M4209, under individual and HS‐stress conditions. Previously, M4209 was found to exhibit inducible salt tolerance by virtue of improved photosynthesis, ion homoeostasis and transcriptional activation of salt‐responsive pathways (Negi et al. 2020). With our post‐germination stress profiling, we carried out a dual investigation into (1) the effect of HS stress on sugarcane growth and physiology and (2) identification of the key adaptations mediating HS‐stress tolerance in sugarcane.

## 2 Materials and Methods

### 2.1 Plant Material, Growth Conditions and Stress Treatments

The entire study was carried out with 60 d old plants of Co 86032 and M4209, using pot cultures in a green‐house facility at Bhabha Atomic Research Centre, Mumbai, India (Figure 1B). The single‐bud stalk sections (setts) were surface treated by dipping in 0.1% carbendazim solution for 3–4 h followed by drying on blotting sheets. Individual setts were planted in plastic containers (22 × 22 cm, one sett per pot) bearing six small holes at the base, filled with a potting soil:soilrite:sand mix (8:1:1). Post‐emergence, the plantlets were irrigated from the bottom of the containers and supplemented with 3.5 g NPK fertiliser (N‐P_2_O_5_‐K_2_O = 20‐20‐20) thrice, one as a basal dose and remaining two intermittent doses at 25 d and 50 d, post‐plantation. At six‐leaf stage, uniformly‐grown plants were organised into four independent sets (Figure 1C): Set‐1 plants were continuously irrigated with half‐strength Murashige and Skoog medium and maintained under ambient temperature conditions (Control); Set‐2 plants were subjected to NaCl stress by incrementally saturating soil starting with 50 mM NaCl solution (prepared in half‐strength Murashige and Skoog medium) with final concentration of 200 mM, and maintained under ambient conditions (NaCl) (Figure 1C); and Set‐3 plants were continuously irrigated with half‐strength Murashige and Skoog medium. To simulate day‐time heat waves, the chamber temperature was daily raised from 30°C to 42°C at 9 AM in the morning using overhead infrared lamps (ramp rate 0.2°C min^−1^), and returned to 30°C at 5 PM in the evening (heat) (Figure 1B‐D). The selection of 42°C for heat stress was based on the average maximum summer temperatures of major Indian sugarcane‐producing states which are co‐affected with salinity (Figure 1A) as well as previously published studies (Gomathi et al. 2013, 2014; Panta et al. 2022). Set‐4 plants were simultaneously subjected to NaCl stress and heat waves (HS) (Figure 1B‐D). At 15 d post‐stress, plant phenotyping was carried out and morphometric indices, including whole shoot dry biomass (g), leaf area and root dry biomass (g) were estimated. Thereafter, the first fully‐expanded leaf from shoot apex was harvested for the quantification of physio‐biochemical markers, expression profiling and liquid chromatography‐mass spectrometry (LC‐MS) based metabolome analysis.

### 2.2 Quantification of Stomatal Attributes

For acquiring epidermal impressions of the abaxial leaf surfaces, nail varnish was applied on the central section of the youngest fully‐emerged leaves at three independent positions and the epidermal peels were transferred onto microscope slides. The stomatal complexes were visualised using light microscopy (Olympus‐IX73) and stomatal density and complex size were quantified as described in Xiong and Flexas (2020). Stomatal density (SD; µm^−2^) was determined as the mean number of stomata per stage, while stomatal complex size (SCS; µm^−2^) was defined as an ellipse with major axis equal to guard cell length and minor axis equal to the width of the entire stomata (20X field of view; 50 µm scale). The stomatal pore dimensions (stomatal pore length [SPL], stomatal pore width [SPW] and stomatal pore area [SPA]), were visualised using scanning electron microscopy of dried leaves, as described in Majumdar et al. (2019). Briefly, the mid‐sections from the first fully‐expanded leaves were sun‐dried, sectioned using microtome and visualised under field emission scanning electron microscope (SEM) (Zeiss SUPRA 55VP). The SEM images were processed using ImageJ software (Schneider et al. 2012).

### 2.3 Estimation of Na^+^ and K^+^ Content

The Na^+^ and K^+^ content in first fully‐expanded leaves were quantified as per Negi et al. (2020). Briefly, oven‐dried leaf samples were digested in conc. HNO_3_ overnight and heated at 120°C till the complete evaporation. The residues were dissolved in demineralised water by vortexing and centrifuged. Clear extracts were analysed using the atomic absorption spectrophotometry (Welsch 1985). The Na^+^ and K^+^ concentrations were expressed in µg g^−1^ DW.

### 2.4 Estimation of Proline and Total Soluble Sugar (TSS) Accumulation

Proline‐accumulation in leaf tissues was quantified by using the acid‐ninhydrin reagent method (Bates et al. 1973) with slight modifications as per Negi et al. (2020). Briefly, 100 mg of frozen leaf tissue was ground in Liq. N_2_, and mixed with aqueous sulfosalicylic acid (3% w v^−1^), followed by centrifugation at 10 000*g* for 10 min at RT. To set up the reaction, 1 mL of this supernatant was mixed with equal volumes of glacial acetic acid and ninhydrin reagent and the reaction mix was boiled for 1 h. The reaction was terminated by snap‐chilling, and allowed to return to RT. Thereafter, the sample was vigorously mixed with 2 mL toluene to allow phase separation and extraction of chromophore in toluene. The latter was aspirated from the aqueous phase and its absorbance was determined at 520 nm using toluene as blank. A standard curve of known proline concentrations was generated and proline content was expressed in μg g^−1^ FW. TSS accumulation in leaf tissues was quantified as per the protocol outlined in Negi et al. (2020). TSS content was expressed in mg g^−1^ FW.

### 2.5 Quantification of Malondialdehyde (MDA) Content and Antioxidant Enzyme Assays

The accumulation of MDA equivalents in leaf tissues was quantified as an indicator of oxidative stress, as per the protocol outlined by Negi et al. (2020). Total MDA content was expressed in μmol L^−1^ g^−1^ FW. For assay of antioxidant enzymes activities in leaf samples, approx. 100 mg of frozen tissues were homogenised in chilled extraction buffer containing 100 mM potassium phosphate buffer (pH 7.0), 0.1 mM ethylenediaminetetraacetic acid (EDTA) and 1% polyvinyl pyrrolidone (PVP) (w v^−1^). The total protein concentrations of the extracts were quantified using the Bradford method (Bradford 1976). The respective activities of superoxide dismutase (SOD), ascorbate peroxidase (APX), guaiacol peroxidase (GPX), catalase (CAT) and glutathione reductase (GR) were quantified as described by Beauchamp and Fridovich (1971), Nakano and Asada (1981), Hemeda and Klein (1990), Aebi (1974) and Smith et al. (1988), respectively and expressed as specific activity (Units mg^−1^ protein).

### 2.6 LC‐MS Based Untargeted Metabolite Profiling

Metabolite extraction and LC‐MS based untargeted metabolite profiling was carried out in accordance with Bansal et al. 2023. Briefly, 100 mg leaf tissues were ground in liquid N_2_ and powdered samples were homogenised in methanol (80% LC‐MS grade) by vortexing for 10 min at room temperature. The mixture was sonicated for 20 min, followed by centrifugation at 13 000 rpm for 10 min. The supernatant was filtered through 0.2 µm syringe filter and used for untargeted LC‐MS‐based metabolite profiling, as described in Bansal et al. (2023). Thereafter, the complete data was uploaded on XCMS online (https://xcmsonline.scripps.edu/) for further untargeted metabolite analysis, including pair‐wise comparison for each treatment set with control set. After filtering the entries on the basis of *p* < 0.05 and log2FC > 2, the metabolites were identified on the basis of several features, including potential adducts in positive ionization mode, m/z values of features, and an experimental mass error (< 10 ppm), along with the information available in databases, including METLIN (https://metlin.scripps.edu/) and LIPIDMAPS (https://www.lipidmaps.org/). An additional stringency cut‐off log2FC > 3 was applied to identify the key differentially accumulated metabolites (DAMs) in each metabolic cluster (NaCl, heat and HS‐stress, normalized with respect to control) for both genotypes. The venn diagrams and sparse partial least squares discriminant analysis (sPLSDA) plots were generated using Venny 2.0.2 and Metaboanalyst 5.0 (https://www.metaboanalyst.ca/), respectively.

### 2.7 Measurement of Photosynthetic and Gas‐Exchange Parameters

Photosynthetic and gas‐exchange parameters were analysed in the first fully expanded leaf of three randomly selected plants from each treatment at 15 DPS, using the GFS‐3000 portable photosystem (Walz, Germany) with the manufacturer's instructions (Supporting Information: Methods S1). Since the average photosynthetic photon flux density (PPFD) in the greenhouse used for plant growth ranged in 800–850 μmol m^−2^ s^−1^, the photosynthetic measurements were carried out at 800 μmol m^−2^ s^−1^ PPFD. The parameters associated with measurement of photosynthetic efficiency were as follows: Cuvette area: 8 cm^2^; cuvette volume: 40 mL, chamber temperature: 42°C/30°C, cuvette air flow: 750 mL min^−1^, relative humidity: 60% and atmospheric CO_2_ concentration: 400 ppm. For measurement of photosynthetic parameters, the leaves were enclosed in the GFS‐3000 leaf chamber, and first acclimated to 100 μmol m^−2^ s^−1^ PPFD until steady‐state net assimilation rate (A_Net_) and stomatal conductance for H_2_O (g_sw_) were visibly reached, after which PPFD was raised to 800 μmol m^−2^ s^−1^ and kept steady for 15 min. Leaves were maintained in their original positions during photosynthesis measurements and the temperature of leaves, cuvette and plant growth chamber was maintained at 42°C/30°C during measurements. The photosynthetic and gas exchange parameters were calculated as specified for GFS‐3000 by the manufacturer (Supporting Information: Methods S1). The total chlorophyll and carotenoid content of leaf tissues was extracted, as described in Srivastava et al. (2006). The pigment contents were calculated as per the formula given by Lichtenthaler (1987).

### 2.8 Expression Profiling of Stomatal Regulators and Photosynthesis‐Related Genes

The expression profiling of stomatal development‐ and photosynthesis‐associated genes was carried out as described in Srivastava et al. (2014), with minor modifications. DNA‐free total RNA was extracted using Tri reagent (T3934, Sigma) and quality was determined based on absorbance values (A260/A280 ratio = 1.8–2 and A260/A230 ratio = 2–2.2) and rRNA band integrity on denaturing agarose gel. Subsequently, cDNA synthesis was carried out using TransScript® First‐Strand cDNA Synthesis SuperMix (Transgen biotech, AT301‐02) following the manufacturer's protocol. The qPCR reaction was set up by combining 10 μL of 2×TransStart® Tip Green qPCR SuperMix (Transgen biotech, AQ141‐02) with 2.5 μL of 1:3 diluted cDNA templates, 1.5 μL each of forward and reverse primer (10 mM each), and 4.5 μL of nuclease free deionized water. The qPCR reaction was carried with three biological replicates with three technical replicates for each sample using CFX96 Touch Real‐Time PCR Detection System (Bio‐Rad Laboratories) with the following protocol: 95°C for 5 min; 40 cycles at 94°C for 20 s, 55°C for 30 s, and 72°C for 30 s followed by 72°C for 10 min and melting curve analysis. The expression data was analysed using CFX Maestro™ software (Bio‐Rad Laboratories) and log2FC was calculated using Microsoft Excel. The primers were designed from the last exon region using NCBI primer BLAST. The details of all primers are given in Supporting Information: Table S1.

### 2.9 Measurement of the Activities of Source‐Sink Metabolic Homoeostasis Related Enzymes

Liquid nitrogen ground leaf samples (~300 mg), were extracted using ice cold buffer containing 100 mM chilled 3‐(N‐morpholino)propanesulfonic acid (MOPS) (50 mM; pH 7.5), EDTA (1 mM), MgCl_2_ (15 mM), phenylmethylsulfonyl fluoride (2 mM) and PVP (2% w v^−1^)]. Following centrifugation (12 000 *g*, 15 min, 4°C), the supernatant was used for measuring the specific activities of SPS (sucrose phosphate synthase) and FBPase (fructose‐1,6‐bisphosphatase).

SPS activity was measured as per the protocol described in Mirajkar et al. (2016). Briefly, 35 µL of the supernatant was mixed with equal volume of a reaction buffer containing 100 mM MOPS NaOH (pH 7.5), 4 mM fructose 6‐phosphate, 20 mM glucose 6‐phosphate, 5 mM MgCl_2_, 1 mM EDTA and 3 mM uridine diphosphate glucose (UDPG). An individual set devoid of UDPG served as the enzyme blank for the reaction. Both the sets were incubated for 60 min in water bath at 37°C. The reaction was terminated by adding 70 µL 30% KOH to the reaction mix, followed by 10 min incubation in a boiling water bath. After cooling the tube contents, sucrose was quantified with the help of freshly prepared anthrone reagent (comprised of 150 mg of anthrone, 76 mL of H_2_SO_4_ and 30 mL of distilled water). For this, 1 mL of anthrone reagent was added to 140 µL of reaction mixture, followed by incubation at 37°C for 20 min. Subsequently, absorbance of the reaction mix was recorded at 650 nm and the amount of sucrose produced was measured by calculating ∆OD (test OD − blank OD) and expressed as specific activity (Units mg^−1^ protein min^−1^) of SPS.

FBPase activity was measured by coupling FBPase‐mediated fructose 6‐phosphate production, with NADP^+^ reduction with phosphoglucose isomerase and glucose 6‐Phosphate dehydrogenase, as described by Lázaro et al. (1974). To initiate the reaction, 0.3 µM NADP^+^ was added to a reaction mixture [50 mM Tris HCl (pH 8), 0.6 µM fructose 1, 6 bis‐phosphate, 1.2 units of phosphoglucose isomerase, 0.6 units of glucose 6‐ phosphate dehydrogenase, and sample aliquot]. The absorbance of the reaction mixture was spectrophotometrically measured at 340 nm and the rate kinetics of the increase in absorbance was used to calculate the specific activity of FBPase (molar extinction coefficient: 6.3 × 10^3^ M^−1^ cm^−1^), expressed in Units mg^−1^ protein min^−1^.

### 2.10 Labelled ^14^C Sucrose Loading Assay

The phloem loading capacity of ^14^C‐labelled sucrose was quantified, as described by Yadav et al. (2017). Briefly, freshly cut mid‐leaf sections were submerged with 2‐(N‐morpholino)ethanesulfonic acid (MES)–CaCl_2_ buffer (20 mM MES, 2 mM CaCl_2_, pH 5.5 adjusted with KOH). Leaf discs (3.6 × 1.0 cm) from these sections were immediately placed (abaxial side down) in a 24‐well cell culture plate containing 1 ml MES–CaCl_2_ buffer containing sucrose solution (1 mM unlabelled sucrose supplemented with 0.81 µCi mL^−1 14^C sucrose). Leaf discs were vacuum‐infiltrated for 30 min with gentle shaking at 25°C. Labelled leaf discs were transferred to a fresh plate and washed twice with 1.0 mL MES–CaCl_2_ buffer. The discs were blot‐dried, frozen on dry ice and lyophilised before the ^14^C sucrose loading capacity was quantified with a scintillation counter (Perkin Elmer 4910TR Tricarb Liquid Scintillation Counter).

### 2.11 Statistical Analysis

Except expression profiling, all data was analysed using SPSS 10.0 (SPSS Inc., Chicago, IL, USA). Before conducting the analysis of variance (ANOVA), the Shapiro‐Wilk test was used to test for validity of normality assumption and variance homogeneity was checked through Levene's test. After the criteria of ANOVA were met, we performed a three‐way ANOVA (NaClxHeatxGenotype) with a balanced design (2 × 2 × 2) to determine the presence of interaction effects between the three independent variables (*p* < 0.05). The statistical significance derived from the descriptive statistics was represented as [*F* (degrees of freedom, value of *F* statistic), *p* = value]. Two‐tailed Student's *t*‐test and correlation analysis was performed using MS‐Excel. For multivariate analysis, all variables were standardised to unit scale (mean = 0 and standard deviation = 1) and principal component analysis (PCA) was performed using Origin (v. 2018).

## 3 Results

### 3.1 M4209 Exhibits Improved Plant Growth Under Combined NaCl and Heat Stress

**Figure 2:**
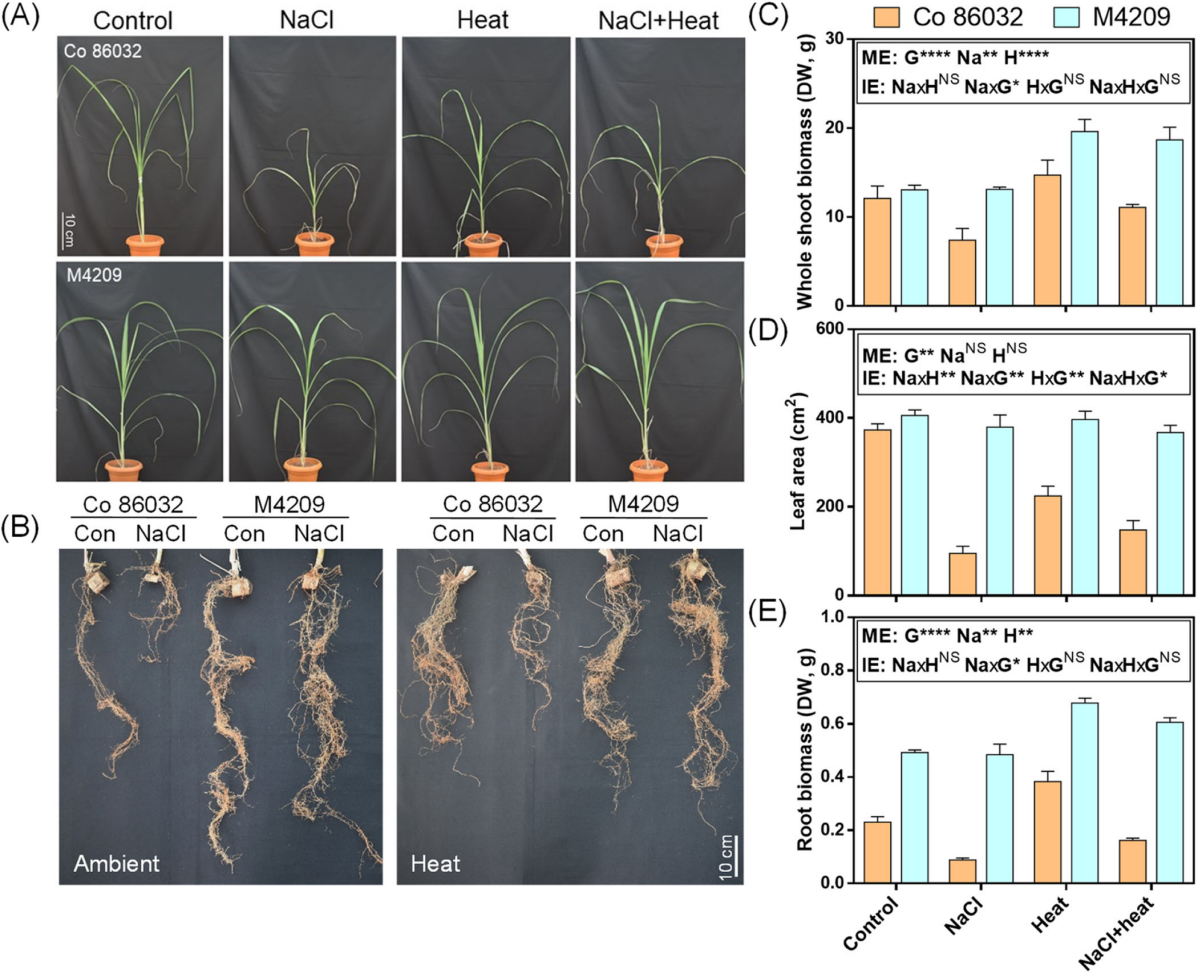
M4209 exhibits improved growth phenotype under combined NaCl and heat stress. Whole shoot phenotype (A), root phenotype (B), whole shoot biomass (C), leaf area (D) and root biomass (E) of 60 d old Co 86032 and M4209 plants under control, NaCl, heat and HS‐stress conditions, respectively. Values are presented as means ± standard error of three independent replicates. Three‐way analysis of variance (ANOVA) (NaClxHeatxGenotype) was used to determine the statistical significance underlying the main effect of NaCl, heat and genotype (denoted by ME) as well as their two‐ and three‐way interaction effects (denoted by IE) on tested parameters at *p* < 0.05. Asterisks indicate the degree of significance [*(*p* ≤ 0.05); [**(*p* ≤ 0.01); ***(*p* ≤ 0.001); ****(*p* ≤ 0.0001)]. [Color figure can be viewed at wileyonlinelibrary.com]

Under control condition, both genotypes exhibited similar shoot biomass and leaf area, but M4209 showed 2.35‐fold higher root biomass than Co 86032 (Figure 2). While M4209 maintained its plant growth (Figure 2A,B) and biomass under NaCl stress (Figure 2C,E), Co 86032 plants exhibited 0.61‐ and 0.45‐fold reduction in shoot and root biomass, respectively, compared with control (Figure 2C,E). This highlighted the genotype‐specificity in NaCl‐induced reduction in shoot [*F* (1, 5.761), *p* = 0.029] and root biomass [*F* (1, 4.613), *p* = 0.047] (Figure 2C,E). By contrast, heat improved both biomass traits irrespective of genotype [shoot: *F* (1, 3.595), *p* = 0.076; root: *F* (1, 0.655), *p* = 0.43], with Co 86032 and M4209 exhibiting 1.21‐ and 1.50‐fold increase in shoot biomass, and 1.62‐ and 1.39‐fold increment in root biomass respectively, over the corresponding controls (Figure 2C,E). In HS‐stressed Co 86032 plants, shoot biomass was found to be comparable (Figure 2C), but root biomass was reduced to 0.92‐fold, relative to control (Figure 2E). By contrast, shoot and root biomass of HS‐stressed M4209 plants were increased by 1.42‐ and 1.24‐fold, respectively, compared with control (Figure 2C,E). While M4209 did not exhibit any significant change in leaf area across the tested stress conditions, the leaf area of Co 86032 plants was reduced by 0.27‐, 0.60‐ and 0.39‐fold, under NaCl, heat and HS‐stress, respectively, compared with control (Figure 2D). Moreover, NaCl and heat independently exerted their contrasting effects on shoot [*F* (1, 0.001), *p* = 0.97] (Figure 2C) and root biomass [*F* (1, 0.037), *p* = 0.850] (Figure 2E), but leaf area was determined by a genotype‐specific interaction between the two stresses [*F* (1, 14.317), *p* = 0.002] (Figure 2D).

### 3.2 M4209's Stomatal Attributes Exhibit Stress‐Responsive Plasticity

**Figure 3:**
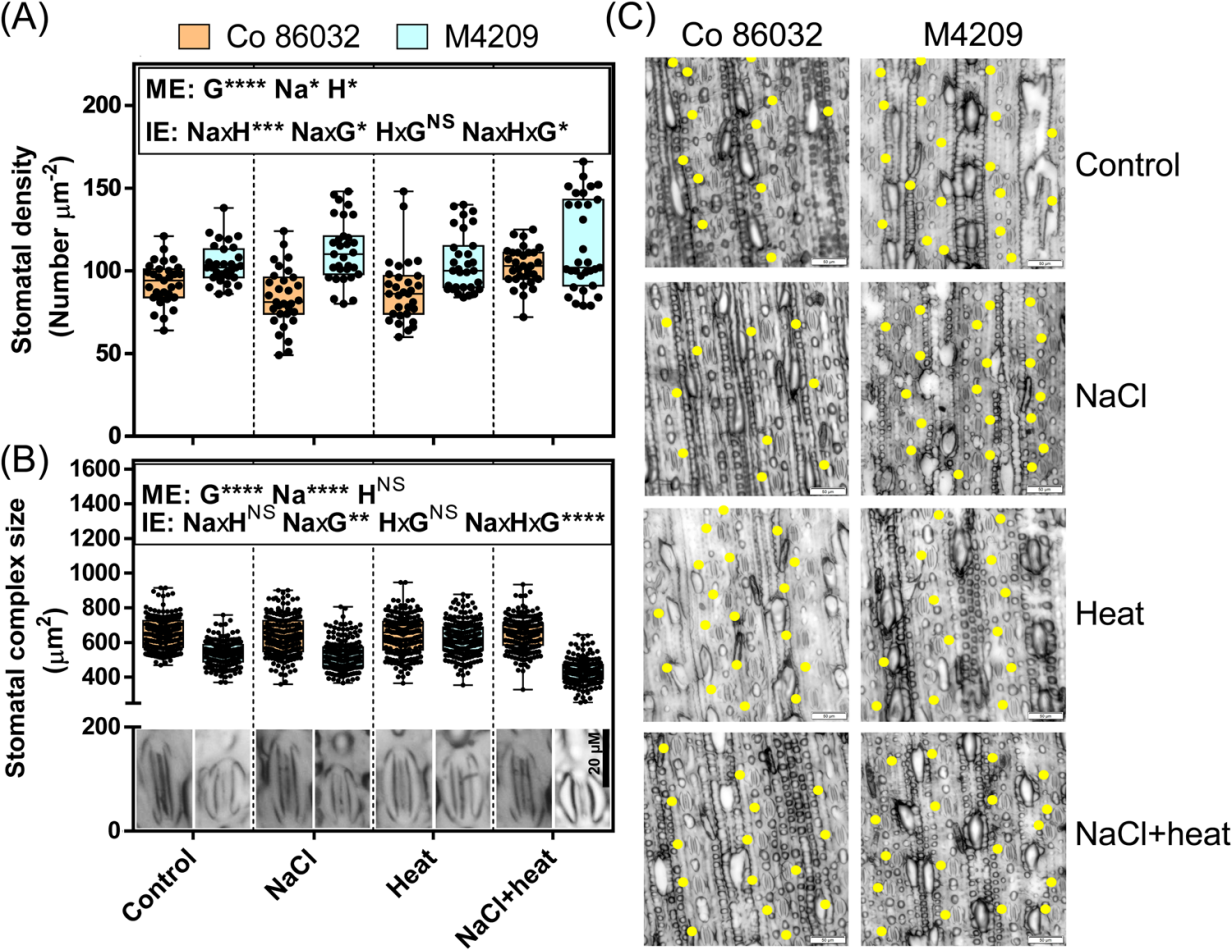
M4209 exhibits OSD (open small dense) stomatal phenotype. Stomatal density (A), Stomatal complex size (B), and stomatal distribution within the epidermal impressions (C), of the first fully‐expanded leaves of Co 86032 and M4209 plants under control, NaCl, heat and heat‐salt (HS)‐stress conditions, respectively. Values are presented as means ± standard error of three independent replicates. Three‐way analysis of variance (ANOVA) (NaClxHeatxGenotype) was used to determine the statistical significance underlying the main effect of NaCl, heat and genotype (denoted by ME) as well as their two‐ and three‐way interaction effects (denoted by IE) on tested parameters at *p* < 0.05. Asterisks indicate the degree of significance [*(*p* ≤ 0.05); [**(*p* ≤ 0.01); ***(*p* ≤ 0.001); ****(*p* ≤ 0.0001)]. [Color figure can be viewed at wileyonlinelibrary.com]

Since stomata mediate gas exchange and water relations in plants and are modulated in response to both heat and NaCl stress, we assessed key stomatal attributes of the abaxial leaf surfaces. Compared to Co 86032, M4209 showed 1.12‐fold higher stomatal density (Figure 3A,C) but 0.83‐fold smaller stomatal complexes (Figure 3B, inset). Further, M4209 exhibited 1.33‐, 1.19‐ and 1.12‐fold higher stomatal density than Co 86032 under NaCl, heat and HS‐conditions, respectively (Figure 3A,C), suggesting that genotype significantly influenced stomatal density [*F* (1, 50.68), *p* = 1.34E‐11]. NaCl stress only increased stomatal density in M4209, underscoring the effect of genotype [*F* (1, 4.222), *p* = 0.041]. On the other hand, heat did not significantly affect stomatal density in either genotype [*F* (1, 2.695), *p* = 0.12] and differentially modified the impact of NaCl by increasing stomatal density in both genotypes [*F* (1, 5.569), *p* = 0.031] (Figure 3A,C). Concomitant with the stress‐responsive increment in stomatal density, M4209 showed 0.82‐ and 0.67‐fold smaller stomatal complex size than Co 86032 under NaCl and HS conditions, respectively, with heat equalising these differences (Figure 3B). By contrast, stomatal complex size remained unchanged in Co 86032 across the tested stress conditions (Figure 3B, inset). Similar to stomatal density, genotype had a significant impact on stomatal complex size [*F* (1, 50.951, *p* = 0.000002]. Further, heat stress modified the impact of NaCl stress in a genotype‐specific manner [*F* (1, 8.778, *p* = 0.0091]. Thus, M4209 exhibited a higher density of small‐sized stomata than Co 86032, and these altered stomatal attributes exhibited stress‐responsive plasticity (Figure 3).

### 3.3 Molecular Regulators of Stomatal Development Are Differentially Expressed in M4209

**Figure 4:**
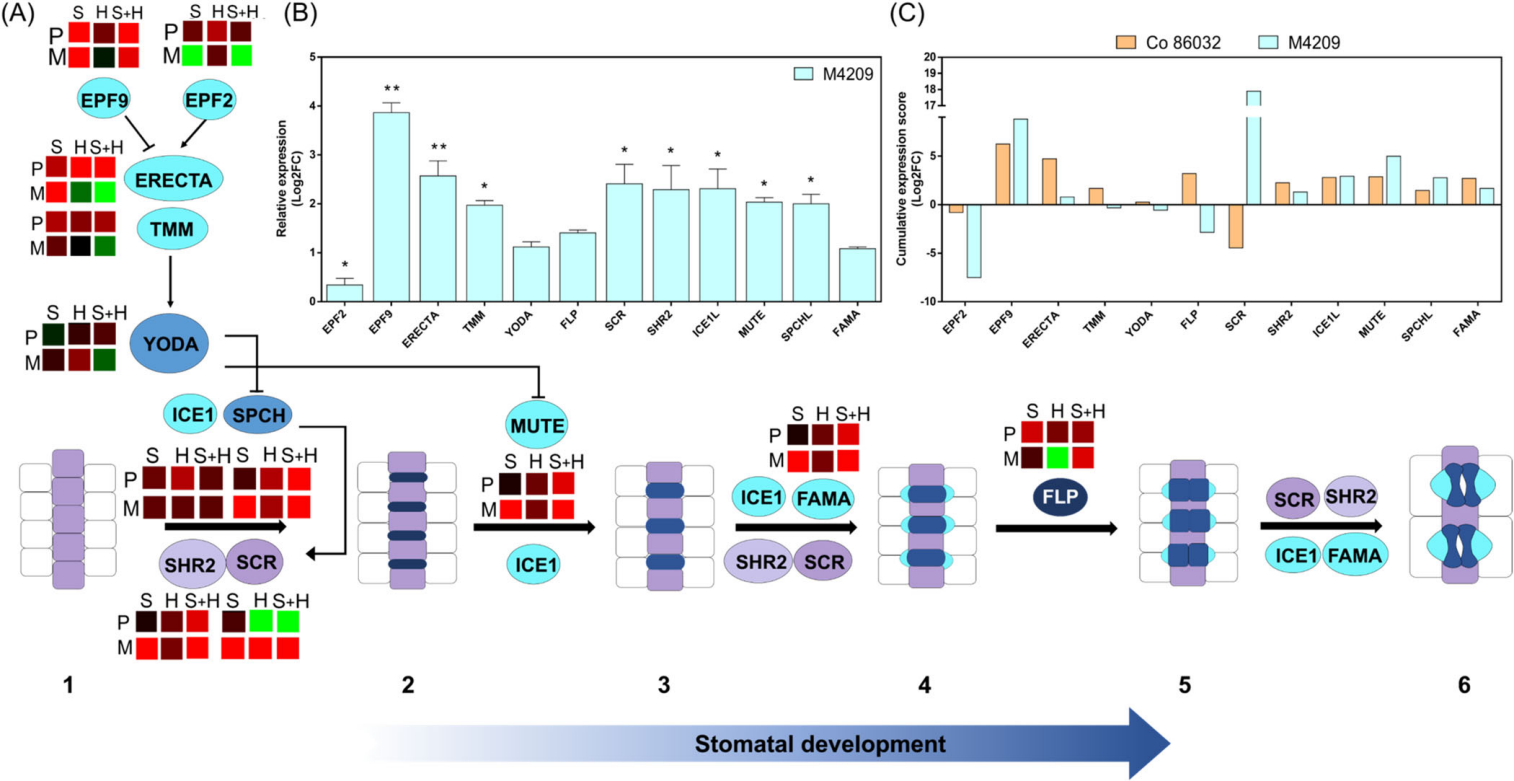
Stomata developmental regulators are differentially expressed in M4209. Summary of stomatal developmental pathway in M4209 under tested stress conditions (A); Protodermal cells which are entering the proliferation phase are termed meristemoid mother cells (MMCs) (violet) (1), MMCs divide into a meristemoid (navy blue) and stomatal‐lineage ground cell (SLGC) (light purple), under the regulation of SPCH/ICE1 and SCR/SHR2. SPCH induces the expression of SCR (2). These meristemoids then transition into guard mother cell (GMC) (blue) as determined by MUTE/ICE1 (3). FAMA/ICE1 and SCR/SHR2 promote the asymmetric division of GMC‐induced subsidiary mother cell (SMC) (light blue) (4). FLP regulates the orientation of GMC division (5). FAMA/ICE1 and SCR/SHR2 regulate the establishment of guard cell (GC) (blue) and subsidiary cell (SC) (light blue) (6). A peptides‐MAP regulatory module regulates the stomatal development. EPF9 (STOMAGEN) and EPF2 act as positive and negative regulators of stomatal development, respectively, via their interaction with the ERECTA‐TMM receptors. The latter activates the YODA kinase which destabilises SPCH and MUTE. Circle colour indicates the particular step of cell specification under their regulation, based on Wu et al. (2019). The heatmaps indicate the differential expression of stomatal development regulators under salt (S, NaCl), heat (H) and salt+heat (S + H) stresses, respectively. The relative expression of genes involved in stomatal development in M4209, under control conditions (B). Asterisks indicate the degree of significance using student's *t*‐test. Cumulative expression score of stomatal regulatory genes across the stress treatments (C), in Co 86032 and M4209 plants, respectively. *EPF*, epidermal patterning factor; *FLP*, four lips; *ICE1*, inducer of CBF expression1; *SCR*, scarecrow; *SHR*, short root; *SPCH*, speechless; *TMM*, too many mouths. [Color figure can be viewed at wileyonlinelibrary.com]

To identify the key stomatal regulators associated with M4209's differential stomatal phenotype, we carried out the expression profiling of a panel of regulatory genes with positive (*epidermal patterning factor 9 [SoEPF9*], *speechless [SoSPCH]*, short root 2 *[SoSHR2]*, *SoMUTE*, *SoFAMA, scarecrow [SoSCR], inducer of CBF 1 [SoICE1], SoERECTA* and *too many mouths [SoTMM*]) and negative *(SoEPF2*, *SoYODA*, and *four lips [SoFLP]*) roles during stomatal development (Figure 4). Interestingly, the native expression of *SoSCR*, *SoSHR2*, *SoMUTE*, *SoFAMA* and *SoICE1*, was doubled in M4209 relative to Co 86032, while SoEPF*9 and SoEPF2* showed 4‐fold and 0.2‐fold higher expression in M4209, relative to Co 86032 (Figure 4B). Under NaCl stress, both *SoSCR* (22.6‐fold) and *SoERECTA* (3.18‐fold) were significantly upregulated in M4209, contrary to the downregulation [*SoSCR* (─4.2‐fold); *SoERECTA* (─0.4‐fold)] observed in Co 86032 plants (Figure 4A). Under heat stress, *SoSCR* (7.8‐fold) *and SoFLP* (─3‐fold) were majorly up‐ and downregulated genes in M4209, respectively, with a opposite trend observed in Co 86032 [*SoSCR* (0.59‐fold); *SoFLP* (1.6‐fold)] (Figure 4A). Under HS‐stress, a complex expression pattern was observed in both the genotypes, which includes both similar (*SoSPCHL*, *FLP* and *EPF2*) and contrasting trend (*SoMUTE*, *SoSCR*, *SoERECTA*, *SoEPF9*, *SoSPCHL* and *SoSHR2*) (Figure 4A). Hence, we attempted to identify the key gene(s) on the basis of cumulative expression score across all the tested stress regimes. In Co 86032, *SoEPF9* (6.24), *SoMUTE* (2.87) and *SoSCR* (─4.46) were identified as prominent determinants of stomatal phenotype on a transcriptional level. On the other hand, *SoEPF2* (─7.5), *SoSCR* (17.9), *SoEPF9* (8.81) and *SoMUTE* (5) emerged as the major transcriptional regulators associated with differential stomatal phenotype in M4209 (Figure 4C).

### 3.4 ‘Open‐Small‐Dense’ Stomata Drive Better Gas‐Exchange and Transpirational Cooling in M4209

**Figure 5:**
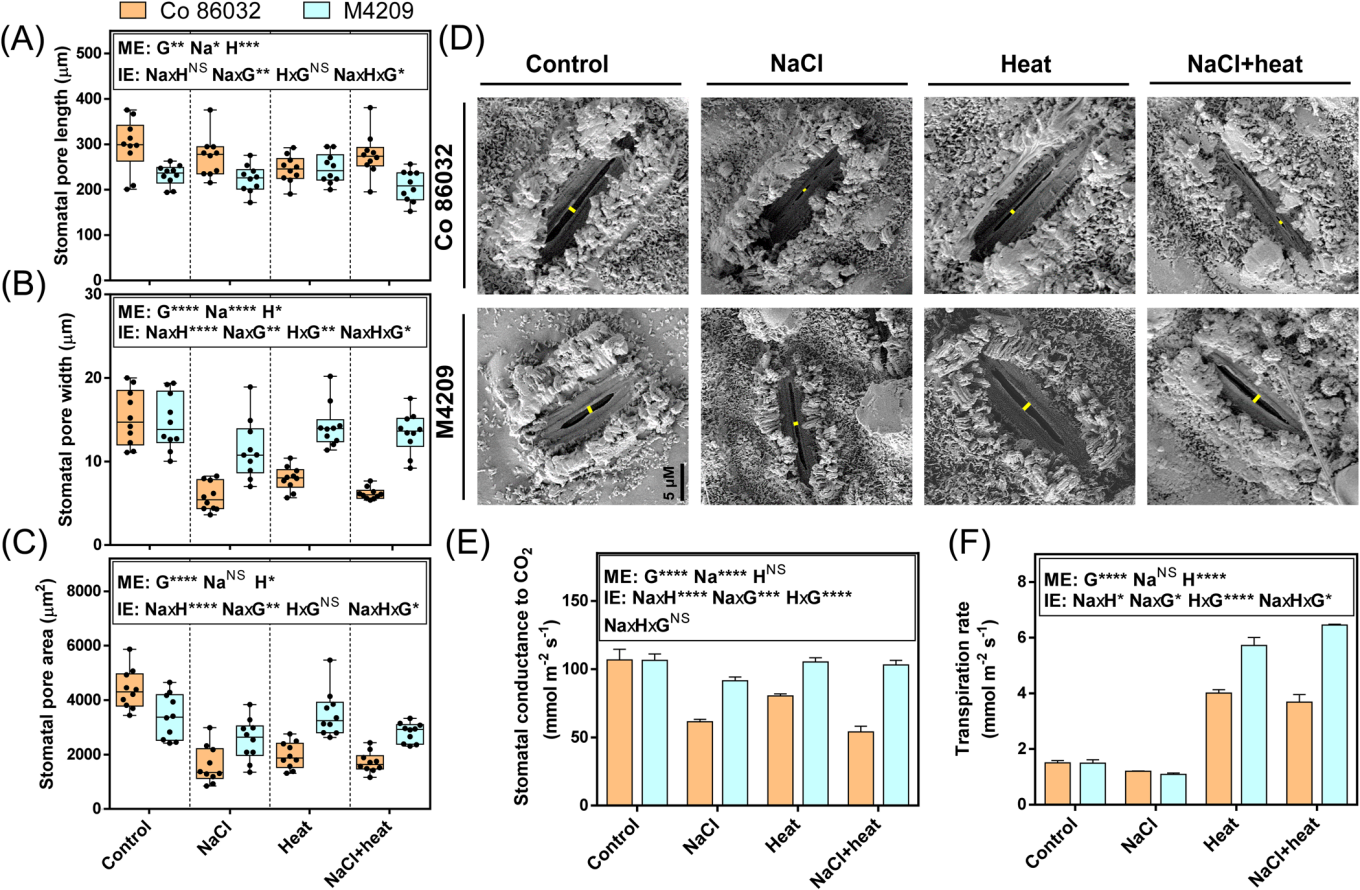
OSD stomata mediate gaseous influx and transpirational cooling in M4209. Stomatal pore length (A), stomatal pore width (B), stomatal pore area (C), SEM‐images of stomatal pore state (D), stomatal conductance to CO_2_ (g_CO2_) (E) and transpiration rate (F) in the first fully‐expanded leaves of Co 86032 and M4209 plants under control, NaCl, heat and heat‐salt (HS)‐stress conditions, respectively. Values are presented as means ± standard error of three independent replicates, unless specified otherwise. Three‐way analysis of variance (ANOVA) (NaClxHeatxGenotype) was used to determine the statistical significance underlying the main effect of NaCl, heat and genotype (denoted by ME) as well as their two‐ and three‐way interaction effects (denoted by IE) on tested parameters at *p* < 0.05. Asterisks indicate the degree of significance [*(*p* ≤ 0.05); [**(*p* ≤ 0.01); ***(*p* ≤ 0.001); ****(*p* ≤ 0.0001)]. SEM, scanning electron microscope; OSD, Open Small Dense. [Color figure can be viewed at wileyonlinelibrary.com]

After characterising the differential stomatal phenotype and associated molecular regulators in M4209, we next attempted to quantify the aperture dimensions (Figure 5). In M4209 stomatal pore length was inherently 0.78‐fold shorter than Co 86032, and this size reduction was consistently observed under both NaCl stress (0.82‐fold) and HS‐stress (0.75‐fold), except heat (Figure 5A), suggesting a strong impact of genotype [*F* (1, 11.718), *p* = 0.003]. Further, the stomata in both genotypes exhibited similar stomatal width (Figure 5B), but 0.77‐fold lower stomatal pore area was observed in M4209 compared with Co 86032, under control conditions. (Figure 5C,D). Further, M4209 showed 1.98‐, 1.79‐ and 2.18‐fold wider stomatal pores under NaCl, heat and HS‐stress conditions, compared to Co 86032 (Figure 5B). As with stomatal pore length, genotype was a strong determinant of stomatal pore width [*F* (1, 29.998), *p* = 0.000051] and also affected the positive impact of heat [*F* (1, 6.11), *p* = 0.025]. While NaCl reduced stomatal width in both genotypes [*F* (1, 4.418), *p* = 0.052] (Figure 5B), this effect was modified by heat application irrespective of genotype [*F* (1, 2.321), *p* = 0.147]. Further, the stomatal pore area in M4209 was 1.58‐, 1.78‐ and 1.64‐fold higher than those of Co 86032 under NaCl, heat and HS conditions, respectively (Figure 5C,D). NaCl stress had an overall negative impact on stomatal pore area, irrespective of genotype [*F* (1, 3.594), *p* = 0.076185], and this effect was countered by heat application but only in M4209 [*F* (1, 9.766), *p* = 0.0065] (Figure 5C,D). We termed the term OSD (open small dense) to describe M4209's stomatal phenotype (Figure 5D).

To determine the impact of these differential stomatal responses on leaf transpiration and CO_2_ influx, we quantified the transpiration rate and g_CO2_ under the tested stress scenarios. While the two genotypes showed similar g_CO2_ under control conditions, exposure to NaCl, heat and HS‐stress reduced g_CO2_ in Co 86032 plants by 0.67‐, 0.54‐ and 0.53‐fold, respectively (Figure 5E). In contrast, only NaCl‐stressed M4209 plants showed significant reduction in g_CO2_, with 1.1‐, 1.7‐ and 2.1‐fold higher g_CO2_ than Co 86032 under NaCl, heat and HS‐stress conditions, respectively (Figure 5E), highlighting the strong impact of genotype on g_CO2_ [*F* (1, 58.53), *p* = 9.85E‐07]. While the effect of both heat [*F* (1, 11.862), *p* = 0.003] and NaCl [*F* (1, 18.957), *p* = 0.000492] was subject to genotype, heat counteracted the negative impact of NaCl stress on g_CO2_ in both genotypes [*F* (1, 0.985), *p* = 0.336]. Further, in both genotypes, transpiration rate of NaCl‐stressed plants was reduced to 0.7‐fold of the rates exhibited by control plants (Figure 5F); in contrast, heat caused transpiration rate to be increased by 2.6‐ and 3.82‐ fold in Co 86032 and M4209, respectively, indicating that the influence of heat was genotype‐dependent [*F* (1, 108.843), *p* = 0.000] (Figure 5F). Under HS‐stress, transpiration rate in Co 86032 and M4209 was found to be increased by 2.45‐ and 4.31‐fold, respectively (Figure 5F), suggesting that heat significantly counteracted NaCl's effect, and this interaction varied across the two genotypes [*F* (1, 6.854), *p* = 0.019].

### 3.5 Heat Stress Affects Root‐To‐Shoot Na^+^ Translocation Under Well‐Watered Conditions

**Figure 6:**
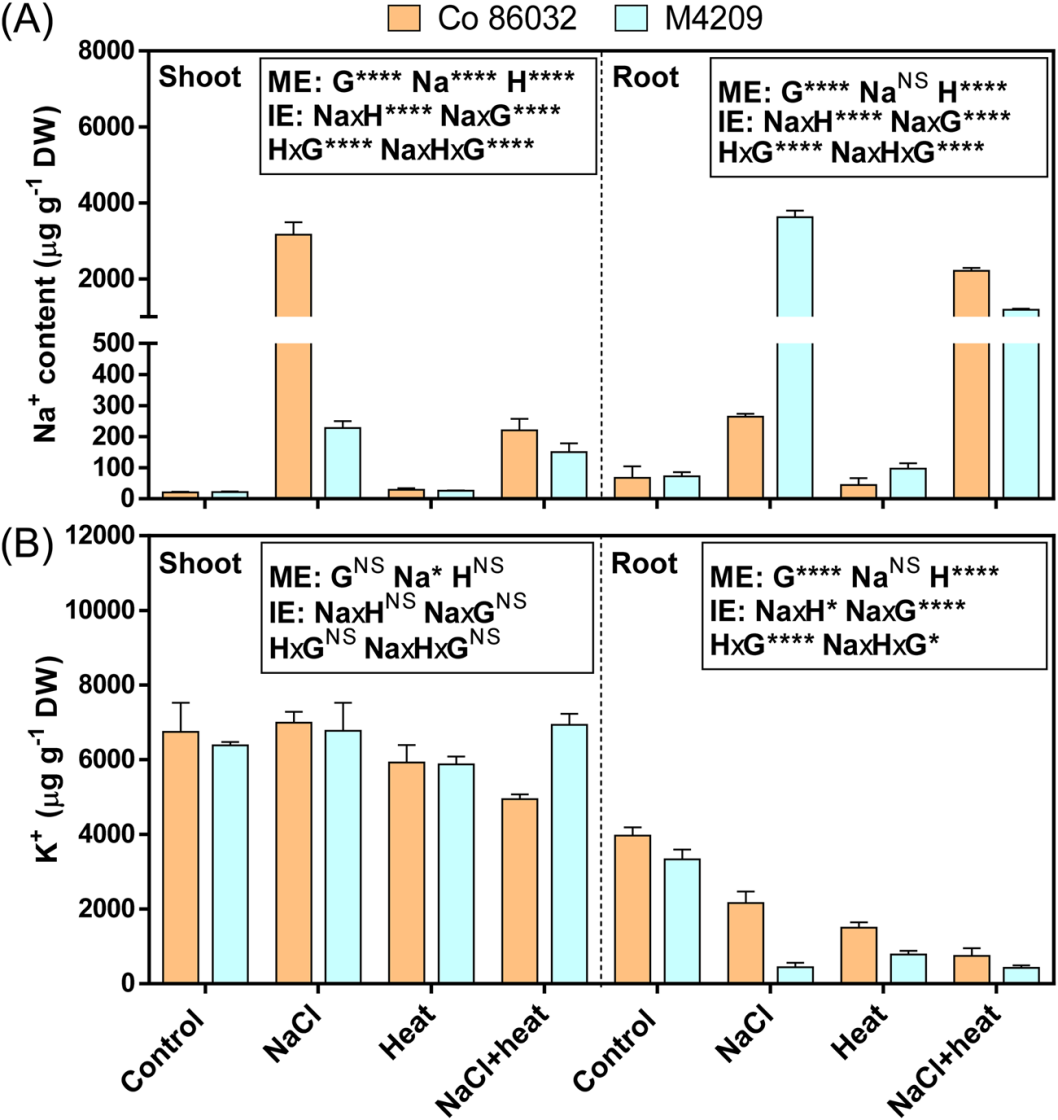
Heat stress reduces root‐to‐shoot Na^+^ translocation. Na^+^ ‐(A) and K^+^ ‐accumulation (B) in the shoots (left) and roots (right) of Co 86032 and M4209. Three‐way analysis of variance (ANOVA) (NaClxHeatxGenotype) was used to determine the statistical significance underlying the main effect of NaCl, heat and genotype (denoted by ME) as well as their two‐ and three‐way interaction effects (denoted by IE) on tested parameters at *p* < 0.05. Asterisks indicate the degree of significance [*(*p* ≤ 0.05); [**(*p* ≤ 0.01); ***(*p* ≤ 0.001); ****(*p* ≤ 0.0001)]. [Color figure can be viewed at wileyonlinelibrary.com]

To investigate the impact of differential stomatal responses on the ion homoeostasis under the tested stress conditions, we quantified Na^+^ and K^+^ accumulation in the third leaf and root tissues. In NaCl‐stressed Co 86032 plants, Na^+^ accumulation was drastically increased by 155.86‐fold, with a corresponding increment of 10.65‐fold in M4209, compared to their respective controls. By contrast, the root Na^+^ accumulation was increased by 3.65‐ and 8.36‐fold in NaCl‐stressed Co 86032 and M4209, respectively (Figure 6A). HS‐stress reduced Na^+^ accumulation in Co 86032 leaves to 0.069‐fold of that observed under NaCl stress, with a modest reduction of 0.66‐fold in corresponding M4209 plants. However, root Na^+^ accumulation was increased and decreased by 8.36‐ and 0.32‐fold in Co 86032 and M4209, respectively, compared to corresponding NaCl‐stressed counterparts (Figure 6A). The impact of NaCl stress on Na^+^ accumulation was genotype‐dependent in shoots [*F* (1, 79.894), *p* = 1.2784E‐07], but not in roots [*F* (1, 1.301), *p* = 0.271]. Further, heat significantly modulated the effect of NaCl stress on Na^+^ accumulation in both leaves [*F* (1, 72.957), *p* = 2.347E‐07] and roots [*F* (1, 104.738), *p* = 1.9886E‐08], in a genotype‐specific manner (Figure 6A).

Under NaCl stress, there was no significant change in the leaf K^+^ accumulation in the two genotypes, however the root K^+^ content was reduced to 0.54‐ and 0.13‐fold in Co 86032 and M4209 respectively, compared to the levels observed in corresponding control plants. (Figure 6B). Heat application reduced the shoot K^+^ levels to 0.87‐ and 0.92‐fold of control levels in Co 86032 and M4209, respectively, with a corresponding reduction in root K^+^ levels by 0.37‐ (Co 86032) and 0.23‐fold (M4209). HS‐stress also reduced the shoot K^+^ levels in Co 86032 plants by 0.73‐ and 0.71‐fold, respectively, compared with respective control and NaCl‐stressed plants, with no significant changed observed in M4209 (Figure 6B). The root K^+^ levels were decreased to 0.19‐ and 0.34‐fold in Co 86032 plants subjected to HS‐stress, compared with the levels observed in control and NaCl‐stressed plants. In M4209, root K^+^ levels were reduced to 0.13‐fold under HS‐stress, relative to control, however, no significant change was observed with respect to NaCl‐stressed plants (Figure 6B). Overall, NaCl stress did not have a significant impact on shoot K^+^ levels [*F* (1, 0.0309), *p* = 0.586], and its effect on root K^+^ levels was heat‐dependent [*F* (1, 42.411), *p* = 7.15991E‐06]. As with Na^+^, heat emerged as a major determinant of K^+^ accumulation in shoots [*F* (1, 6.327), *p* = 0.023] and modified the effect of NaCl stress in roots [*F* (1, 7.148), *p* = 0.017], in a genotype‐specific manner (Figure 6B).

### 3.6 Heat Dominates NaCl Stress in Terms of Biochemical Responses

We also evaluated the impact of single and combined stress regimes on oxidative stress markers, osmolyte accumulation and antioxidant defence in the two genotypes. NaCl evoked greater biochemical response which was most evident in Co 86032, as seen for 3‐fold higher MDA levels (Supporting Information: Figure S2A), 112‐fold higher proline (Supporting Information: Figure S2B), 11‐fold higher SOD activity (Supporting Information: Figure S3B), and 7‐fold higher catalase (Supporting Information: Figure S3C), compared with control. On the other hand, M4209 showed 0.35‐fold lower MDA levels (Supporting Information: Figure S2A), with activation of catalase (5.5‐fold; Supporting Information: Figure S3C) and GR (5.27‐fold; Supporting Information: Figure S3D) activities being the major NaCl‐specific biochemical response, relative to control. On the other hand, heat stress only triggered the activation of APX (5‐fold; Supporting Information: Figure S3A) and SOD (3.5‐fold; Supporting Information: Figure S3B) in Co 86032, relative to control, while other parameters showed minor changes. Under HS‐conditions, heat dominated the effect of NaCl on all parameters in a genotype specific manner [MDA: *F* (1,12.874), *p* = 0.0026; APX: *F* (1, 27.904), *p* = 0.000074; GR: *F* (1, 5.733), *p* = 0.029; SOD: *F* (1, 6.529), *p* = 0.021; proline: *F* (1, 4.529), *p* = 0.049; catalase: *F* (1, 9.899), *p* = 0.006; TSS: *F* (1, 5.619), *p* = 0.031] (Supporting Information: Figure S2,S3). Except for APX and catalase activities in HS‐stressed M4209, all other parameters in both genotypes were comparable to their respective levels under heat stress. Both genotypes showed similar TSS levels under the tested conditions, with a minor reduction observed in control M4209 plants, relative to Co 86032. (Supporting Information: Figure S2C).

### 3.7 Genotype and Stress‐Specific Lipid Remodelling in Sugarcane

**Figure 7:**
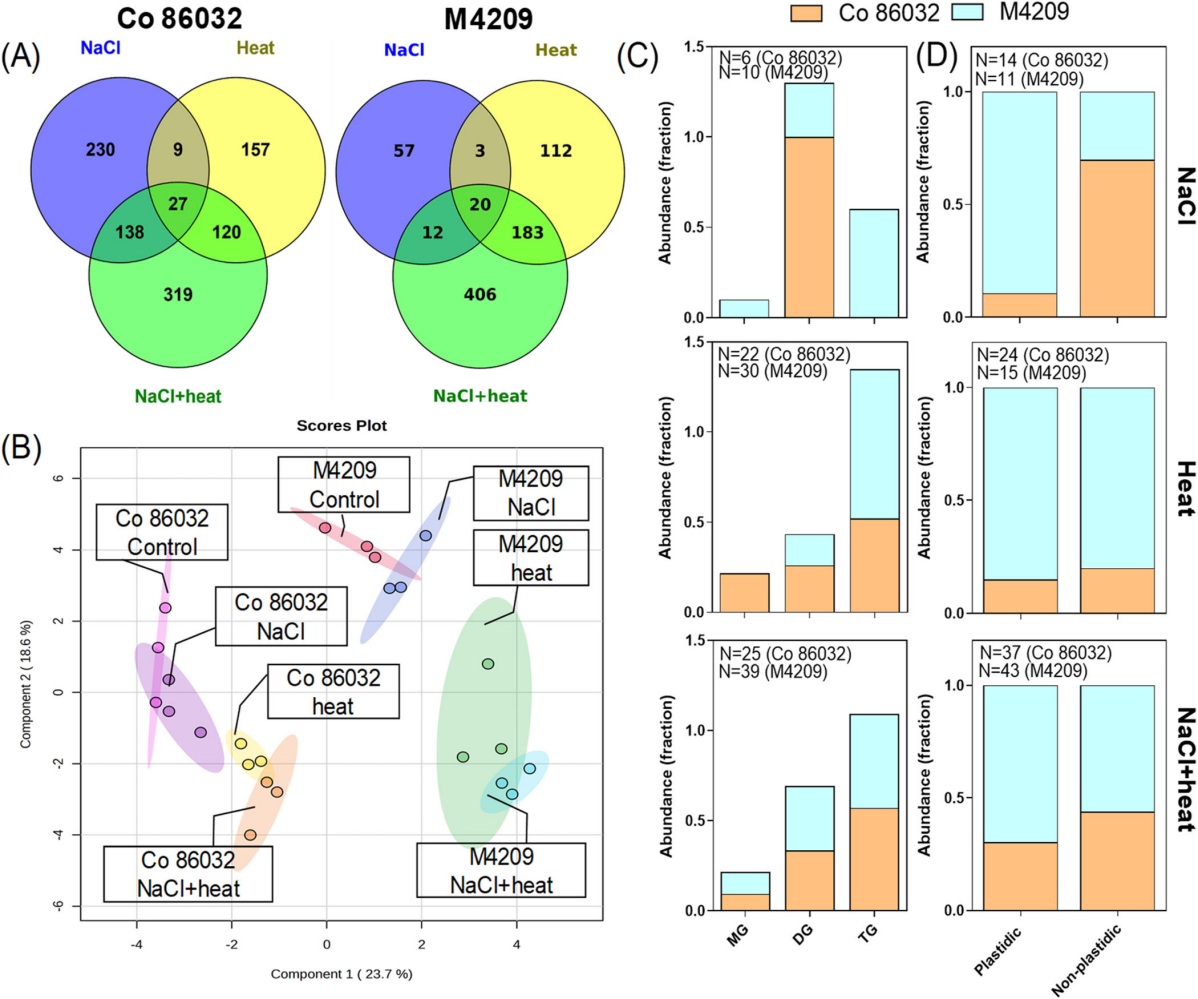
M4209 exhibits differential stress‐specific accumulation of triacylglycerols (TGs) and plastidic lipids. Venn diagram depicting the distribution of DAMs (differentially accumulated metabolites) identified through liquid chromatography‐mass spectrometry (LC‐MS)‐based metabolite profiling in the first fully‐expanded leaves of Co 86032 and M4209 plants (A), sPLSDA‐based clustering of DAMs (B) along with abundance ratios of mono‐ (MG), di‐(DG) and tri‐acylglycerols (TG) (C) and plastidic:non‐plastidic phospholipids (D) in the stress‐wise metabolic clusters of Co 86032 and M4209 under NaCl, heat and NaCl + heat stress. [Color figure can be viewed at wileyonlinelibrary.com]

Non‐targeted metabolome profiling of the first fully‐expanded leaves indicated that NaCl‐stressed Co 86032 plants accumulated 4.39‐fold higher DAMs than corresponding M4209 plants, with a comparable number of DAMs in the heat and HS‐clusters of both genotypes (Figure 7A). The sPLSDA‐based clustering showed that in both the genotypes, HS cluster overlapped with heat, while NaCl cluster overlapped with control (Figure 7B). Among the annotated DAMs, lipids emerged as the major organised metabolite class, with two distinct trends (Figure 7C,D). While the Co 86032‐NaCl cluster solely consisted of diacylglycerols (DGs), TGs were most prominent species in M4209‐NaCl cluster, followed by diacylglycerols (DGs) and monoacylglycerols (MGs) (Figure 7C, top panel). Co 86032‐heat cluster showed the same order of abundance (TG > DG > MG); however, no MG was detected in M4209 (Figure 7C, middle panel). Under HS stress, both genotypes showed a similar trend (TG > DG > MG) with comparable abundance of each component (Figure 7C, bottom panel). The phospholipid composition of the two genotypes also exhibited a distinct trend across the tested stress conditions. In M4209‐NaCl cluster, the abundance ratio of plastidic:non‐plastidic phospholipids shifted towards the plastidic fraction (0.9:0.1), contrary to Co 86032 (0.3:0.7) (Figure 7D, top panel). Although heat equalised this difference between the two genotypes (Figure 7D, middle panel), HS‐stressed M4209 plants exhibited a greater abundance of plastidic lipids than Co 86032 (Figure 7D, bottom panel).

### 3.8 M4209 Harnessed Heat‐Induced Photosynthetic Enhancement via Better Biochemical and Metabolic Integrity

**Figure 8:**
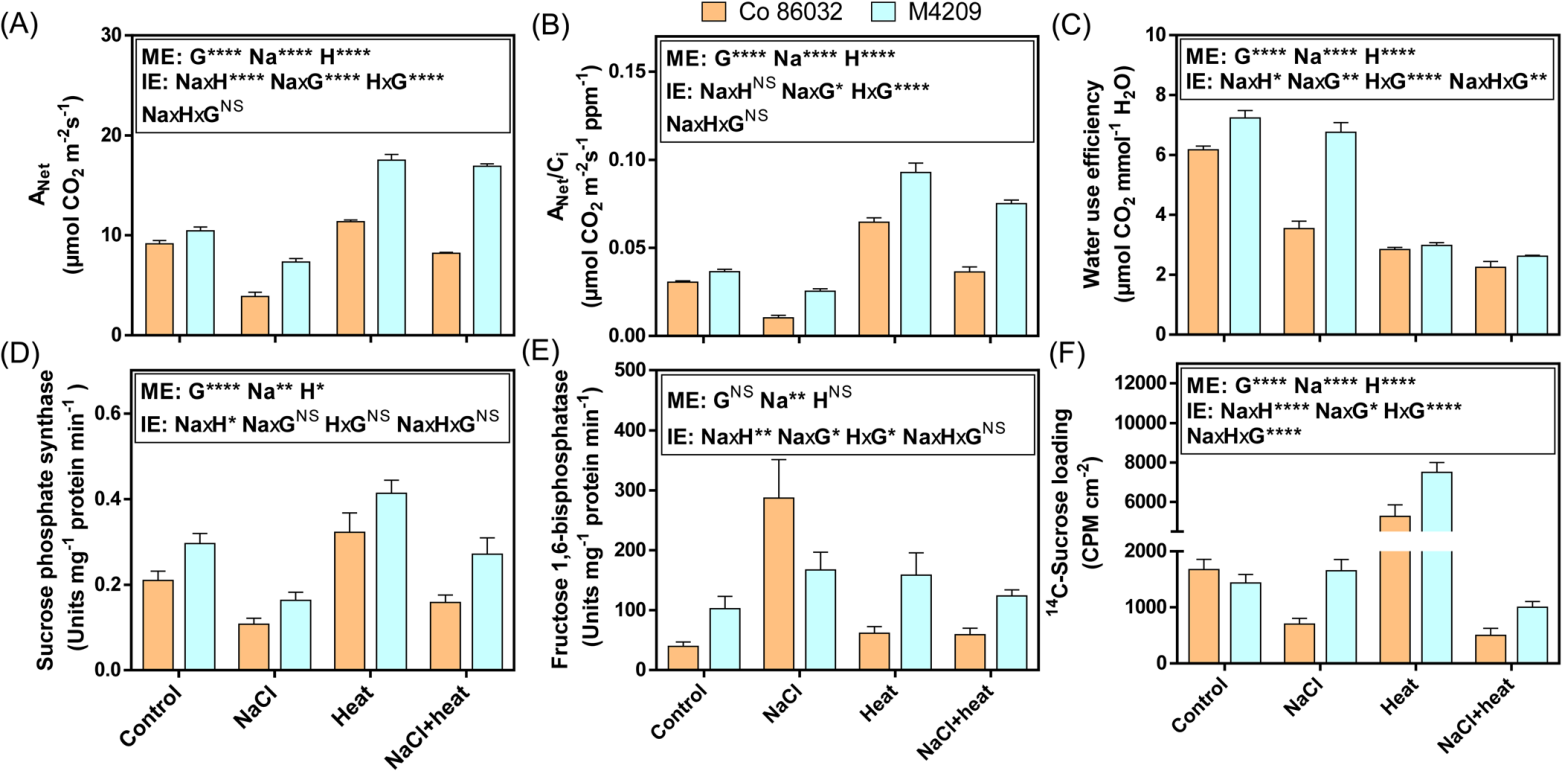
M4209 exhibits significantly improved photosynthetic assimilation, source strength and sucrose transport. A_Net_ (Net photoassimilation rate) (A), A_Net_/C_i_ (instantaneous carboxylation efficiency) (B), water use efficiency (C), sucrose phosphate synthase (SPS) activity (D), FBPase activity (E) and ^14^C‐sucrose loading (F) in the first fully‐expanded leaves of Co 86032 and M4209 plants under control, NaCl, heat and HS‐stress conditions, respectively. Values are presented as means ± standard error of three independent replicates. Three‐way analysis of variance (ANOVA) (NaClxHeatxGenotype) was used to determine the statistical significance underlying the main effects of NaCl, heat and genotype (denoted by ME) as well as their two‐ and three‐way interaction effects (denoted by IE) on tested parameters at *p* < 0.05. Asterisks indicate the degree of significance [*(*p* ≤ 0.05); [**(*p* ≤ 0.01); ***(*p* ≤ 0.001); ****(*p* ≤ 0.0001)]. [Color figure can be viewed at wileyonlinelibrary.com]

Both genotypes exhibited similar A_Net_ under control conditions (Figure 8A). In Co 86032, A_Net_ was reduced by 0.42‐ and 0.89‐fold under NaCl and HS‐stress, and increased by 1.24‐fold under heat stress, compared with control (Figure 8A). Except the comparable A_Net_ under heat and HS‐stress, a similar trend was observed in M4209, such that 1.88‐fold, 1.51‐ and 2.1‐fold higher than Co 86032 under NaCl, heat and HS‐stress (Figure 8A). While the effects of both heat [*F* (1, 11.08), *p* = 0.004] and NaCl [*F* (1, 12.186), *p* = 0.003] were genotype‐dependent, heat modified the effects of NaCl stress in both the genotypes [*F* (1, 4.28), *p* = 0.055]. In contrast to the trend observed in g_CO2_ (Supporting Information: Figure 5E), Co 86032 had higher C_i_/C_a_ than M4209 under both NaCl and HS‐stress (Supporting Information: Figure S5A). While, M4209 exhibited 1.2‐fold higher instantaneous carboxylation efficiency (A_Net_/C_i_) than Co 86032 under control conditions, it showed 2.5‐, 1.44‐ and 2.08‐fold increment under NaCl, heat and HS‐stress conditions (Figure 8B). The higher A_Net_ in M4209 was linked to the higher PSII yield (Supporting Information: Figure S5B) and electron transport rate (Supporting Information: Figure S5C), across the stress conditions. However, unlike A_Net_, the interaction of heat and NaCl was genotype‐dependent for both PSII yield [*F* (1, 11.08), *p* = 0.004] and electron transport rate [*F* (1, 11.08), *p* = 0.004]. Interestingly, the positive correlation was observed between A_Net_ and stomatal density (*r* = 0.95 for Control, NaCl and heat; 0.85 for HS; *p:* 0.0013) across all the tested conditions. M4209 inherently exhibited a 1.5‐fold higher WUE than Co 86032 and this remained unchanged under NaCl stress, contrary to the 0.53‐fold reduction observed in NaCl‐stressed Co 86032 plants, compared with respective controls (Figure 8C). On the other hand, heat‐induced increase in transpiration rate reduced WUE similarly in both genotypes under heat stress, though M4209 still showed 1.2‐fold higher WUE than HS‐stressed Co 86032 plants (Figure 8C). Thus, heat significantly modified the effect of NaCl stress, in a genotype‐specific manner [*F* (1, 9.92), *p* = 0.0062]. Only NaCl and HS‐stress had a significant effect on photosynthetic pigments in the two genotypes, with NaCl‐stressed Co 86032 showing lowest pigment content (Supporting Information: Figure S6A,B). Contrary to Co 86032, the maintained expression/differential upregulation of photosynthesis‐related genes, including *SoRCA* (Rubisco activase), *SoPSBS* (PSII small subunit), *SoRBCS* (Rubisco small subunit) and *SoOEP3* (Oxygen evolving protein 3) in M4209 across the stress conditions further highlighted the superior integrity of its photosynthetic apparatus (Supporting Information: Figure S6C).

### 3.9 Better Source Strength and Sucrose Loading Facilitated M4209's Improved Growth Under HS‐Stress

To ascertain if the improved performance of M4209 under the tested stress regimes was due to the differential modulation of source‐strength and phloem loading, we measured the specific activities of SPS and FBPase, as well as ^14^C‐based phloem loading in the two genotypes (Figure 8D‐F). In Co 86032, SPS activity was found to be reduced by 0.59‐, 1.51 and 0.76‐fold, respectively under NaCl, heat and HS‐stress conditions (Figure 8D). Although a similar trend was observed in M4209, it showed 1.36‐fold higher SPS activity than Co 86032 under control conditions itself, and maintained similar increment over Co 86032 under NaCl (1.4‐fold), heat (1.34‐fold) and Hs‐stress (1.65‐fold) (Figure 8D). Compared to heat [*F* (1, 18.512), *p* = 0.000548], NaCl [*F* (1, 35.104), *p* = 0.000021] had a greater impact on SPS activity, which was also affected by genotype [*F* (1, 15.05), *p* = 0.00133]. By contrast, FBPase activity increased in Co 86032 by 7.21‐, 1.54 and 1.48‐fold, respectively, under NaCl, heat and HS‐stress conditions (Figure 8E). M4209 also showed 2.57‐fold higher FBPase activity than Co 86032 under control conditions, and maintained this increment under heat and HS‐stress (Figure 8E). However, FBPase activity in NaCl‐stressed M4209 plants was 0.6‐fold lower than Co 86032 counterparts (Figure 8E). In Co 86032, NaCl stress reduced ^14^C sucrose loading by 0.47‐fold [*F* (1, 1243.582), *p* = 1.3388E‐16] while heat stress increased it by 3.28‐fold over control plants [*F* (1, 686.645), *p* = 1.4342E‐14]. While M4209 also showed a similar trend, it maintained 2.19‐ and 1.44‐ higher ^14^C sucrose loading than Co 86032 under NaCl and heat stress, respectively (Figure 8F). Under combined stress, the genotype‐dependent antagonistic interaction between heat and NaCl stress reduced ^14^C sucrose loading in both Co 86032 (0.32‐fold) and M4209 (0.69‐fold) [*F* (1, 70.588), *p* = 2.9203E‐07], however M4209 showed 1.97‐fold higher ^14^C sucrose loading than Co 86032 in absolute terms (Figure 8F).

## 4 Discussion

The present study deals with the comparative analysis of Co 86032 (salt‐sensitive parent) and M4209 (salt‐tolerant mutant derived from Co 86032), to understand the effect of HS‐stress on sugarcane, in terms of physiology and associated adaptive responses. Although the differential root biomass was observed as major phenotypic distinction between the genotypes, their variable responses became more distinct over the tested stress scenarios (Figure 2). This was especially prominent under NaCl stress, since contrary to Co 86032, M4209 did not exhibit any discernible growth or biomass penalty (Figure 2). Earlier, transcriptional reprogramming of stress‐responsive pathways and improved photosynthetic efficiency were associated with the inducible salt tolerance trait of M4209 (Negi et al. 2020). Short‐term heat waves significantly improved overall growth and biomass traits in both the tested sugarcane genotypes, with greater biomass gains realised in M4209 (Figure 2A‐C,E). This contradicts the heat‐induced reduction in plant growth normally observed in seed‐propagated temperate or C3 plants (Moore et al. 2021). Such a thermo‐morphogenic growth enhancement in vegetatively‐propagated tropical sugarcane could be attributed to warm temperatures accelerating leaf turnover rate and canopy development in sugarcane (Hatfield and Prueger 2015). In addition, our experimental setup entailed night‐time recovery following day‐time heat‐waves (Figure 1) and well‐watered conditions to avoid any interference from drought‐like scenario, which could potentially exacerbate the thermal susceptibility of plant tissues.

Under HS‐stress, the positive influence of heat outweighed the deleterious impact of NaCl stress on plant growth and biomass traits in both the tested genotypes, particularly in Co 86032 (Figure 2A‐C,E). In contrast, the leaf area in M4209 remained unchanged under heat with/without salt stress (Figure 2D), highlighting that genotypic variation played a key role in determining interaction between heat and NaCl stress conditions. Further, in the salt‐sensitive Co 86032, heat equalised the negative impact of NaCl stress in terms of biomass (Figure 2C,E), which was more prominent in shoots. Such heat‐mediated suppression of negative effects of salt stress has also been shown previously in other crops (Rivero et al. 2014; Nahar et al. 2022). In particular, the beneficial effect of heat can be attributed to pronounced transcriptional and metabolite level reprogramming than salt stress, which takes a longer time to induce comparable‐level change, particularly in the aerial tissues (Tan et al. 2023). The relatively higher shoot biomass gain in M4209 under both heat and HS‐stress (Figure 2C), could be attributed to its inherent ability to activate phenylpropanoid/terpenoid pathway, which results in production of flavonoids, terpenoids and soluble phenolics (Supporting Information: Figure S1; Negi et al. 2020). The enhanced accumulation of these compounds has been shown to improve thermotolerance by improving antioxidant defence, membrane stability and thereby support photosynthetic activity and overall plant growth, under high temperature conditions (Liu et al. 2021). In addition, being a salt‐tolerant genotype, M4209 experienced limited perturbation in ionic (Figure 6) and redox homoeostasis (Supporting Information: Figures S2; S3), thus avoiding the diversion of resources towards antioxidant defence (Supporting Information: Figures S2, S3). As a consequence, M4209 was better able to harness the heat‐induced growth benefits under HS‐conditions (Figure 2).

Earlier, gamma irradiation‐based physical mutagenesis was shown to alter stomatal size and density in sugarcane varieties (Yasmeen et al. 2020), with an generally inverse relation between these two traits (Bheemanahalli et al. 2021). In a similar fashion, the gamma irradiation‐derived mutant M4209 exhibited an inherently higher stomatal density (Figure 3A,C), but smaller stomatal complex size than Co 86032 (Figure 3B, inset). Despite the enhanced density, M4209 stomata were organised in a non‐clustered epidermal patterning (Figure 3C), which is unlikely to negatively impact leaf anatomy (McKown and Bergmann 2020). M4209's differential stomatal attributes could be due to the inherent up‐and downregulation of *SoEPF9* and *SoEPF2*, respectively (Figure 4B), which encode for functionally antagonistic signal peptides. While EPF9 promotes the initiation into stomatal lineage to promote stomatal density, EPF2 suppresses excessive meristemoid production and division, resulting in few, large‐sized stomata (McKown and Bergmann 2020). In *Arabidopsis*, the suppression of *AtEPF2* led to dual increment of stomatal density as well as rooting area (Hepworth et al. 2016). Thus, *SoEPF2* could be one of putative causal genetic factors behind the ‘steep‐deep‐heap’ root phenotype of M4209 (Figure 2B,E). In addition, a strong positive correlation (*r* = 0.78) was observed between stomatal density and root growth in M4209, suggesting a hydraulic synergy between root and shoot systems, that is a prerequisite for optimal plant growth (Karlova et al. 2021).

Based on the PCA score, stomatal density was identified as one of the major M4209‐specific determinants of stress resilient phenotype (Supporting Information: Figure S7). The decreased and increased stomatal densities in NaCl‐stressed Co 86032 and M4209 plants, respectively (Figure 3A), recalls a well‐documented contradiction underlying stomatal plasticity for salt‐adaptation. Plants either reduce their stomatal density to restrict water loss at the cost of gaseous influx (Shahzad et al. 2022), as seen in Co 86032 (Figure 3A and 5E,F), or increase stomatal density (Figure 3A) to promote gas‐exchange and maximise photosynthesis (Kiani‐Pouya et al. 2020), similar to M4209 (Figures 5E,F and 8A). However, the positive correlation (*r* = 0.6) between stomatal density and photosynthetic rate of NaCl‐stressed M4209 plants, suggested that a moderate increment in stomatal density might be a successful strategy for improving salt tolerance in sugarcane, as seen earlier (Yasmeen et al. 2020). In heat‐stressed M4209, the coupled up‐ and downregulation of *SoEPF2* and *SoEPF9*, respectively, (Figure 4A,C) resulted in similar stomatal complex sizes between the two genotypes, with little impact on inherent stomatal densities (Figure 3A,B). Upon co‐occurence, both stresses exhibited variable synergy, as stomatal densities were increased in the two genotypes (Figure 3A), but only M4209 exhibited reduced stomatal complex size (Figure 3B, inset). The simultaneous upregulation of *SoEPF9* and *SoMUTE* (Figure 4A,C) could explain the higher stomatal densities in both the tested genotypes, under HS‐stress conditions (Figure 3A). Further, the accentuation of M4209's OSD phenotype under HS‐stress (Figure 3) could be explained on the basis of comparative up‐ and downregulation of *SoSCR* and *SoEPF2*, respectively, relative to control (Figure 4A,C).

In general, small stomata, as seen in M4209, exhibit ‘speedy’ aperture kinetics, since a greater membrane surface area‐to‐volume ratio enables a quicker exchange of ions and solutes between the guard cells and neighbouring epidermal cells (Lawson and Blatt 2014). Under NaCl‐stress, the stomata were largely ‘closed’ (Figure 5B–D), which could potentially limit photosynthetic gas exchange in Co 86032 (Figures 5E and 8A), and vice versa was seen in M4209. Despite this, both genotypes showed comparable transpiration rates under ambient conditions, with or without NaCl stress (Figure 5F). This could be explained on the basis of comparable level of VPD (Supporting Information: Figure S4), which is one of the major drivers of transpiration rate (Grossiord et al. 2020), and hence, masked the impact of differential g_CO2_ on transpiration rates (Figure 5E,F). Further, heat stress induced stomatal pore ‘opening’, and equalised the NaCl‐induced pore closure under HS‐stress conditions in both the genotypes (Figure 5B‐D). These results could be traced to the drastic increase in VPD (Supporting Information: Figure S4), which would trigger stomatal transpiration to compensate for evaporative loss (Urban et al. 2017; Schönbeck et al. 2022), especially under our well‐watered experimental conditions. Such VPD‐responsive transpirational cooling has been identified as a key thermo‐adaptive trait in heat‐tolerant snap‐bean genotypes (Deva et al. 2020). The heat‐induced g_CO2_ and transpiration were more prominent in M4209, and this gap was further pronounced under HS‐stress conditions (Figure 5E,F), that compensated the slightly lower VPD relative to Co 86032 (Supporting Information: Figure S4). Such heat‐responsive transpiration losses (Figure 5F) nullified the higher WUE exhibited by M4209 under ambient conditions (Figure 8C), which underscores the need for further stomatal trait attenuation in light of future climatic scenarios (Lunn et al. 2024).

In M4209, the transpiration rate remained unchanged under NaCl stress, but, was increased under heat stress, which is expected to magnify Na^+^ accumulation in aerial tissues, as shown previously (Suzuki et al. 2016; Jan et al. 2021; Caine et al. 2023). However, contrary to our expectation, NaCl‐stressed M4209 plants showed significantly lower Na^+^ accumulation in shoot than Co 86032 (Figure 6A). Besides, root Na‐accumulation remained higher, indicating reduced root‐to shoot Na^+^ translocation in M4209, which is a well‐known salt‐adaptive trait (Garcia‐Daga et al. 2024). Accordingly, M4209 was better able to circumvent the Na^+^‐induced ionic and oxidative toxicity than Co 86032 (Supporting Information: Figures 6, S2 and S3). Another contributory factor could be the relatively higher accumulation of TGs (Figure 7C), which are the principal constituents of lipid droplets. The latter entrap toxic lipid intermediates resulting from stress‐induced disintegration of lipid membranes, thereby limiting further oxidative damage and conferring tolerance to environmental stresses, including NaCl and heat stress (Lu et al. 2020). Heat‐induced equalised reduction in leaf Na^+^ content and synergistic increase in root Na^+^ content suggested that Co 86032 mimicked M4209‐like root Na^+^‐retention phenotype under HS‐stress conditions. On the other hand, reduced root‐to‐shoot Na^+^‐translocation phenotype was retained in M4209, even under HS‐stress conditions (Figure 6A). We explain these counterintuitive observations to a potential de‐coupling of the stomatal transpiration and long‐distance Na‐transport under heat and salt combination, as seen earlier (Rivero et al. 2014). While the exact mechanisms underlying this decoupling requires further investigation, a possible explanation could be the heat‐induced inactivation of the root‐to‐shoot Na^+^ transporters [HAKs (high‐affinity K^+^ transporter)] and/or activation of Na^+^ extrusion pathways [SOS1 (salt‐overly‐sensitive 1)] (Colmenero Flores and Rosales 2014). Except for HS‐stressed Co 86032 plants, both genotypes largely maintained their leaf K^+^ levels under stress conditions, at the cost of root K^+^ accumulation (Figure 6B). An antagonistic interaction was observed between heat and NaCl stress, in terms of root K^+^ accumulation, that was limited to Co 86032 (Figure 6B). The modulation of ion homoeostasis under heat stress requires further investigation.

While M4209 is a photosynthetically superior genotype than Co 86032 (Negi et al. 2020), the improved photosynthetic parameters under all the tested stress regimes was attributed to lower C_i_/C_a_ ratio (Supporting Information: Figure S5A), indicating better carboxylation efficiency (Figure 8B). Although, high temperature was earlier shown to induce photosynthetic inhibition (Zahra et al. 2023), in the present study, heat stress differentially improved A_Net_ with/without NaCl, with greater increase seen in M4209 than Co 86032 (Figure 8A). Such heat‐mediated photosynthetic gains in M4209 can be explained on the basis of two reasons. In warm‐adapted C4 crops like sugarcane, Rubisco activation requires a higher temperature optimum (Sage et al. 2013), during vegetative development. Further, the relative retention of photosynthetic pigments (Supporting Information: Figure S6A,B), upregulation of key photosynthesis‐related genes like *SoRCA* (Supporting Information: Figure S6C), and higher plastidic:non‐plastidic lipid ratio relative to Co 86032 (Figure 7D) likely contributed towards thylakoid membrane stability and function at high temperatures (Zahra et al. 2023). Besides, SPS activity was reduced, increased and equalised under NaCl, heat and HS‐stress, with M4209 exhibiting comparatively higher SPS activity than across all stress conditions (Figure 8D). Previously, the overexpression of *SoSPS1* improved both sucrose content and biomass production in sugarcane (Anur et al. 2020), suggesting that higher SPS and FBPase activities in M4209, reflects a better source strength (Mehdi et al. 2024). In contrast, lowest SPS but highest FBPase activity indicated carbon reallocation towards starch synthesis in NaCl‐stressed Co 86032 plants (Figure 8E), which can pose a feed‐back limitation to carbon metabolism and plant growth (Mehdi et al. 2024). In line with these observations, M4209 also showed comparatively higher ^14^C sucrose loading under all tested stress conditions, while heat promoted it in both genotypes (Figure 8F), which indicate mobilisation of sugar pool at elevated temperatures (Mehdi et al. 2024). Under HS‐stress conditions, NaCl‐imposed restriction on sucrose loading, prevailed over heat‐induced positive effects (Figure 8F). This suggested that NaCl had a greater impact in coordinating the sucrose export under the tested stress conditions (Perri et al. 2019). PCA scores identified both SPS activity and ^14^C sucrose loading as major contributors to the differential responses exhibited by Co 86032 and M4209 across the stress conditions (Supporting Information: Figure S7), suggesting that better source strength and sucrose transport in M4209 also accounted for improved growth under the tested stress scenarios.

## 5 Conclusions

The present study demonstrated that the sugarcane mutant M4209 retained heat‐induced growth enhancement under HS‐stress conditions. While reduced root‐to‐shoot Na^+^ translocation contributed, the dual increment in stomatal density and photosynthetic assimilation were majorly responsible for M4209's improved performance under combined stress scenario. The increased stomatal density was attributed to the stress‐inducible transcriptional reprogramming of stomatal regulators, specifically *SoSCR* and *SoEPF9*‐*SoEPF2* module. The ‘open’ stomatal pores enabled transpirational cooling and gas exchange necessary for improved photosynthesis in M4209 under all tested stress scenarios. This was further supported by the improved photosystem integrity, owing to better pigment retention, higher plastidic:non‐plastidic lipid ratio and upregulation of key photosynthesis‐associated genes. Unlike many seed crops, the primary commercial product of sugarcane cultivation is sucrose‐filled canes, which demands generally well‐irrigated conditions in commercial sugarcane cultivating areas. Therefore, boosting photosynthesis rather than reducing transpiration could be a viable approach, for better WUE and yield across diverse agro‐climatic regions. Although, being a complex long‐duration, interspecific hybrid crop and due to the random mutagenesis induced by gamma irradiation, the trait‐to‐gene mapping could not be done; however, M4209‐like genotypes having higher stomatal density and photosynthetic assimilation can still be good systems for delineating the effect of combined stresses and associated adaptive traits. The insights gained from the present study can be extrapolated to other crop systems, for both basic as well as applied usage.

## Conflicts of Interest

The authors declare no conflicts of interest.

## Supporting information

Fig. S1: Differential accumulation of terpenoid derivatives and phenolics in M4209 relative to Co 86032 under control conditions. Fig. S2: Oxidative stress marker and osmolyte accumulation in Co 86032 and M4209 under the tested stress scenarios. Fig. S3: Vapour pressure deficit in Co 86032 and M4209 under the tested stress conditions. Fig. S4: Heat suppresses NaCl‐induced activation of antioxidant enzymes. Fig. S5: Differential gaseous influx and photosynthesis‐associated parameters in M4209. Fig. S6: M4209 exhibits greater stability in terms of photosynthesis‐related components. Fig. S7: PCA‐score based identification of key traits contributing towards improved growth of M4209 across the tested stress scenarios.

Supplementary methods: Additional details regarding measurement of gas exchange and photosynthetic parameters under the tested stress conditions.

Table S1: List of all primers used in the study.

## Data Availability

The data that support the findings of this study are available from the corresponding author upon reasonable request.
