# Supplementary material for: Stomata‐Photosynthesis Synergy Mediates Combined Heat and Salt Stress Tolerance in Sugarcane Mutant M4209
Source: Plant Cell Environ. 2025 Mar 7;48(6):4668–84. doi: 10.1111/pce.15424 (PMC12050391; doi:10.1111/pce.15424)
Supplement: Supplementary file 1 — Fig. S1: Differential accumulation of terpenoid derivatives and phenolics in M4209 relative to Co 86032 under control conditions. Fig. S2: Oxidative stress marker and osmolyte accumulation in Co 86032 and M4209 under the tested stress scenarios. Fig. S3: Vapour pressure deficit in Co 86032 and M4209 under the tested stress conditions. Fig. S4: Heat suppresses NaCl‐induced activation of antioxidant enzymes. Fig. S5: Differential gaseous influx and photosynthesis‐associated parameters in M4209. Fig. S6: M4209 exhibits greater stability in terms of photosynthesis‐related components. Fig. S7: PCA‐score based identification of key traits contributing towards improved growth of M4209 across the tested stress scenarios. [file PCE-48-4668-s003.docx]

**Fig. S1: Differential accumulation of terpenoid derivatives and phenolics in M4209 relative to Co 86032 under control conditions.**

**Fig. S2: Oxidative stress marker and osmolyte accumulation in Co 86032 and M4209 under the tested stress scenarios.** Malondialdehyde equivalents (A), proline (B), and total soluble sugars (C), in the first fully-expanded leaves of Co 86032 and M4209 plants under control, NaCl, heat and HS-stress conditions, respectively. Values are presented as means ± standard error of 3 independent replicates. Three-way analysis of variance (ANOVA) (NaClxHeatxGenotype) was used to determine the statistical significance underlying the main effects of NaCl, heat and genotype (denoted by ME) as well as their two- and three-way interaction effects (denoted by IE) on tested parameters at *p*<0.05. Asterisks indicate the degree of significance [*(*p* ≤ 0.05); [**(*p* ≤ 0.01); ***(*p* ≤ 0.001); ****(*p* ≤ 0.0001)].

**Fig. S3: Heat suppresses NaCl-induced activation of antioxidant enzymes.** Specific activities of ascorbate peroxidase (A), superoxide dismutase (B), catalase (C) and glutathione reductase (D), in the first fully-expanded leaves of Co 86032 and M4209 plants under control, NaCl, heat and HS-stress conditions, respectively. Values are presented as means ± standard error of 3 independent replicates. Three-way analysis of variance (ANOVA) (NaClxHeatxGenotype) was used to determine the statistical significance underlying the main effects of NaCl, heat and genotype (denoted by ME) as well as their two- and three-way interaction effects (denoted by IE) on tested parameters at *p*<0.05. Asterisks indicate the degree of significance [*(*p* ≤ 0.05); [**(*p* ≤ 0.01); ***(*p* ≤ 0.001); ****(*p* ≤ 0.0001)].

**Fig. S4: Vapour pressure deficit in Co 86032 and M4209 plants** **under the tested stress scenarios.** Values are presented as means ± standard error of 3 independent replicates. Three-way analysis of variance (ANOVA) (NaClxHeatxGenotype) was used to determine the statistical significance underlying the main effects of NaCl, heat and genotype (denoted by ME) as well as their two- and three-way interaction effects (denoted by IE) on tested parameters at *p*<0.05. Asterisks indicate the degree of significance [*(*p* ≤ 0.05); [**(*p* ≤ 0.01); ***(*p* ≤ 0.001); ****(*p* ≤ 0.0001)].

**Fig. S5: Differential gaseous influx and photosynthesis-associated parameters in M4209.** Ratio of internal to external CO_2_ levels (A), PSII yield (B), and electron transport rate (C), in the first fully-expanded leaves of Co 86032 and M4209 plants under control, NaCl, heat and HS-stress conditions, respectively. Values are presented as means ± standard error of 3 independent replicates. Three-way analysis of variance (ANOVA) (NaClxHeatxGenotype) was used to determine the statistical significance underlying the main effects of NaCl, heat and genotype (denoted by ME) as well as their two- and three-way interaction effects (denoted by IE) on tested parameters at *p*<0.05. Asterisks indicate the degree of significance [*(*p* ≤ 0.05); [**(*p* ≤ 0.01); ***(*p* ≤ 0.001); ****(*p* ≤ 0.0001)].

**Fig. S6: M4209 exhibits greater stability in terms of photosynthesis-related components.** Total chlorophyll levels (A), carotenoid levels (B), and expression profiles of photosynthesis-related genes (C), in the first fully-expanded leaves of Co 86032 and M4209 plants under control, NaCl, heat and HS-stress conditions, respectively. So*TUB* and So*ACT* were used as reference for expression profiling. Values are presented as means ± standard error of 3 independent replicates. Three-way analysis of variance (ANOVA) (NaClxHeatxGenotype) was used to determine the statistical significance underlying the main effects of NaCl, heat and genotype (denoted by ME) as well as their two- and three-way interaction effects (denoted by IE) on tested parameters at *p*<0.05. Asterisks indicate the degree of significance based on Three-way ANOVA or Student’s t-test [*(*p* ≤ 0.05); [**(*p* ≤ 0.01); ***(*p* ≤ 0.001); ****(*p* ≤ 0.0001)]. *SoRBCS*: rubisco small subunit; *SoPSBS*: PSII small subunit; *SoRCA*: rubisco activase; *SoOEP3:* oxygen evolving protein 3; *SoACT*: actin; *SoTUB*: tubulin.

**Fig. S7: PCA-score based identification of key traits contributing towards improved growth of M4209 across the tested stress scenarios.** The input data for each parameter was standardized by subtracting the category mean from the individual datapoint and dividing the difference by standard deviation. PCA was carried out using Origin (v. 2020) and three components (PC1-PC3) were quantified. The PCA scores were derived by summing the products of principle component with the eigenvector values of each parameter along the principal component. The PCA scores were organized from highest to lowest. SPS: Sucrose phosphate synthase activity; ^14^C Suc: ^14^C Sucrose; Na_Root_: root Na^+^ accumulation; Na_shoot_: Shoot Na^+^ accumulation A/C_i_: Instantaneous carboxylation efficiency; TSS: Total soluble sugars; Yield: PSII yield; ETR: Electron transport rate; A_Net_ : Net photoassimilation rate; FBPase: Fructose1,6-bisphosphatase activity; SD: stomatal density; SCS: Stomatal complex size; TR: transpiration rate; SPL: Stomatal pore length; SPA: Stomatal pore area; MDA: Malondialdehyde equivalents; SPW: Stomatal pore width; GPX: Guaiacol peroxidase activity; APX: Ascorbate peroxidase activity; g_CO2_:stomatal conductance to CO_2;_ SOD: Superoxide dismutase activity; GR: glutathione reductase activity; PRL: Proline levels; WUE: Water use efficiency; Ci: Intracellular CO_2_ concentration; Ci/Ca ratio of intracellular to ambient CO_2_ concentration
