# Supplementary material for: Stomata‐Photosynthesis Synergy Mediates Combined Heat and Salt Stress Tolerance in Sugarcane Mutant M4209
Source: Plant Cell Environ. 2025 Mar 7;48(6):4668–84. doi: 10.1111/pce.15424 (PMC12050391; doi:10.1111/pce.15424)
Supplement: Supplementary file 2 — Supplementary methods: Additional details regarding measurement of gas exchange and photosynthetic parameters under the tested stress conditions. [file PCE-48-4668-s001.docx]

**Stomata-photosynthesis synergy mediates combined heat and salt stress tolerance in sugarcane mutant M4209**

**SUPPLEMENTARY METHODS**

***Measurement of gas exchange and photosynthetic parameters*** (in accordance with manufacturer’s instructions)

Transpiration rate (TR, mmol m^−2^ s^−1^]) was calculated as [TR= {Flow∗(dH_2_OMP − dH_2_OZP)}/{Area ∗ (1 − wa)}], wherein,

dH_2_OMP(୲) = H_2_Osam(୲) (H_2_O mole fraction in sample cell of analyzer) − H_2_Oabs(୲) (H_2_O mole fraction in reference cell of analyzer) at measuring point (MP) (ppm)

dH_2_OZP = H_2_Osam (H_2_O mole fraction in sample cell of analyzer) - H_2_Oabs (H_2_O mole fraction in reference cell of analyzer) at zero point (ZP) (ppm)

wa = H_2_Osam − dH_2_OZP (ppm)

Area= Sample area (8 cm^2^)

A_Net_ (µmol m^-2^ s^-1^) was calculated as **[**A_Net_ = {Flow * (dCO_2_ZP − dCO_2_MP)}/Area – (TR * ca)**]**, wherein,

dCO_2_ZP= CO_2_sam(t) [CO_2_ mole fraction in sample cell of analyzer] - CO_2_abs(t) [CO_2_ mole fraction in reference cell of analyzer] at zero point (ZP) (ppm)

dCO_2_MP = CO_2_sam_(t)_ [CO_2_ mole fraction in sample cell of analyzer] - CO2abs_(t-CO2delay_ [CO_2_ mole fraction in reference cell of analyzer, accounting for time delay for gas flowing through the measuring head to reach the analyzer w.r.t. the gas flowing through the reference path] at measuring point (MP) (ppm)

ca [CO_2_ Mole Fraction] = CO_2_sam − dCO_2_ZP (ppm)

Area= Sample area (8 cm^2^)

The instantaneous water use efficiency (WUE, µmol CO_2_ mmol^-1^ H_2_O) was calculated as **[**WUE=A_Net_/TR**]**. The instantaneous carboxylation efficiency (µmol m^−2^ s^−1^ ppm^−1^) was calculated as **[**instantaneous carboxylation efficiency= A_Net_/internal CO_2_ concentration (C_i_)**]**. Vapor-Pressure-Deficit (VPD; Pa) was calculated as **[**VPD = [{SVP(Tleaf)/Pamb} – wa]/1- [{SVP(Tleaf)/Pamb}+wa]/2**]**, wherein

SVP (Tleaf) = saturation vapor pressure at leaf temperature (Tleaf)

Pamb= Ambient pressure (pa)

Wa=H_2_Osam − dH_2_OZP (ppm)

Stomatal conductance to water vapour, g_sw_ (mmol m^-2^ s^-1^) was calculated as **[**g_sw_ = TR/VPD**]**. Stomatal conductance to CO_2_ (g_CO2_, mmol m^-2^ s^-1^) was calculated as **[**g_CO2_=g_sw_/1.6**]**, where 1.6 is the ratio of H_2_O diffusivity to CO_2_ diffusivity in air (Jarvis, 1971). The electron transport rate was determined by applying stepwise increases in the PPFD from 50–800 µM m^−2^ s^−1^ with 5 min at each intensity to achieve stable readings. Quantum yield of photosynthetic electron transport (PSII yield), was quantified as [PSII yield = 1 – (F/Fm′)], wherein,

F= Fluorescence (mV)

Fm’= Fluorescence of the illuminated leaf during a saturating light pulse (mV)

The electron transport rate through PSII was calculated as electron transport rate = **[**PSII yield* (PAR/2) * ETRFac**]**

whereby:

PAR: photosynthetically active radiation

ETR-Fac: PAR absorbed by the sample

**Reference:**

Jarvis, P. 1971. “The Estimation of Resistances to Carbon Dioxide Transfer.” In Plant Photosynthetic Production. Manual of Methods, 566–631. Dr W. Junk NV.
