# Supplementary material for: Stomata‐Photosynthesis Synergy Mediates Combined Heat and Salt Stress Tolerance in Sugarcane Mutant M4209
Source: Plant Cell Environ. 2025 Mar 7;48(6):4668–84. doi: 10.1111/pce.15424 (PMC12050391; doi:10.1111/pce.15424)
Supplement: Supplementary file 3 — Table S1: List of all primers used in the study. [file PCE-48-4668-s002.docx]

**Table S1: List of primers used in the study.** *ICE1*: inducer of CBF expression 1, *ICE1L*: *ICE1*-like; *ERL2*: erecta-like 2; *EPF*: epidermal patterning factor; *SCR*: scarecrow; *SHR*: short root; *SPCH*: SPEECHLESS; *TMM*: too many mouths; *FLP*: four lips; *SoRBCS*: rubisco small subunit; *SoPSBS*: PSII small subunit; *SoRCA*: rubisco activase; *SoOEP3:* oxygen evolving protein 3; *SoACT*: actin; *SoTUB*: tubulin.

| S. no. | Primer name | Primer sequence (5'-3') | Length (bp) | Tm (°C) | GC content (%) |
| --- | --- | --- | --- | --- | --- |
| 1 | SoMUTE_qF | TGCTCCACCTCAACATCAGC | 20 | 60.32 | 55.00 |
| 2 | SoMUTE_qR | GGCCATAGCTGAGGAGTACATC | 22 | 60.03 | 54.55 |
| 3 | SoFAMA_qF | CTGATCAAGACCATCGCTGC | 20 | 59.06 | 55.00 |
| 4 | SoFAMA_qR | TTGACGTCGATGAAGCTGAGG | 21 | 60.40 | 52.38 |
| 5 | SoICE1L_qF | GAAGGGCATGCAGTCAATATCC | 22 | 59.44 | 50.00 |
| 6 | SoICE1L_qR | TACATTGCGTTGTGGAGACCG | 21 | 60.94 | 52.38 |
| 7 | SoTMM_qF | GCTATGACACCAAGCAAGAAGG | 22 | 59.58 | 50.00 |
| 8 | SoTMM_qR | GCTACAAACCATGCAAGGTGC | 21 | 60.67 | 52.38 |
| 9 | SoERL2_qF | TGCTGTGCACCAAACGGC | 18 | 61.90 | 61.11 |
| 10 | SoERL2_qR | TGCTTGGATATGACCTCGCC | 20 | 59.89 | 55.00 |
| 11 | SoYODA_qF | AGGCGCATCAAGTCTCACAG | 20 | 60.39 | 55.00 |
| 12 | SoYODA_qR | TAGCCCAGGGTGTAGAGAGC | 20 | 60.40 | 60.00 |
| 15 | SoEPF2_qF | ATTGCGAGGCAGTGCAGG | 18 | 60.75 | 61.11 |
| 16 | SoEPF2_qR | TTGTAGTTGGAGTGGTCGTCG | 21 | 60.00 | 52.38 |
| 17 | SoEPF9_qF | GTCTAAACAAGGGCATACTGTCG | 23 | 59.38 | 47.83 |
| 18 | SoEPF9_qR | CACCTGTGGCAAACACACTTG | 21 | 60.47 | 52.38 |
| 23 | SoFLP_qF | CCAGCTCACACAGTTCTCCG | 20 | 60.67 | 60.00 |
| 24 | SoFLP_qR | GCCTTGACCCTGGTGAGG | 18 | 59.65 | 66.67 |
| 25 | SoSCR_qF | ACGTCAAGTTCGGCAGCTG | 19 | 60.96 | 57.89 |
| 26 | SoSCR_qR | TGAGCAGGCAGAGGTCCTTC | 20 | 61.55 | 60.00 |
| 27 | SoSHR1_qF | TTCTCTCCGGTGGCGTTC | 18 | 59.35 | 61.11 |
| 28 | SoSHR1_qR | TTGCCCACACGAGAGGC | 17 | 59.60 | 64.71 |
| 29 | SoSHR2_qF | ATTGTGGACCTCGTGTCCTG | 20 | 59.68 | 55.00 |
| 30 | SoSHR2_qR | TTGCTCCTTCCACGCGAG | 18 | 60.05 | 61.11 |
| 31 | SoSPCH_qF | TGGTGGTGAAGACGGTGTC | 19 | 59.56 | 57.89 |
| 32 | SoSPCH_qR | AACGCTCGCTGAATCTCTTG | 20 | 58.65 | 50.00 |
| 33 | SoRCA_qF | CAAGAAGCTCGTCAACTCCAAGG | 23 | 61.65 | 52.17 |
| 34 | SoRCA_qR | TCTTCATGGCGTCCTCGTTGG | 21 | 62.69 | 57.14 |
| 35 | SoOEC3_qF | GTCCAACCTCAAGCAGGATCTG | 22 | 60.94 | 54.55 |
| 36 | SoOEC3_qR | ATCAAGGGCGGAGACGATGTTG | 22 | 62.94 | 54.55 |
| 37 | SoRBCS_qF | ATCAAGAAGTTCGAGACGCTGTC | 23 | 60.92 | 47.83 |
| 38 | SoRBCS_qR | GTAGTGTCTGCCGTCGTTGTAC | 22 | 61.23 | 54.55 |
| 39 | SoPSBS_qF | AACGCCTTCTCCCTCATCG | 19 | 59.48 | 57.89 |
| 40 | SoPSBS_qR | GTGACGAACCTTCCGGTGC | 19 | 61.32 | 63.16 |
| 41 | SoACT_qF | CTGGAATGGTCAAGGCTGGT | 20 | 59.96 | 55.00 |
| 42 | SoACT_qR | TCCTTCTGTCCCATCCCTACC | 21 | 60.34 | 57.14 |
| 43 | SoTUB_qF | CCAAGTTCTGGGAGGTGATCTG | 22 | 60.36 | 54.55 |
| 44 | SoTUB_qR | TTGTAGTAGACGTTGATGCGCTC | 23 | 60.97 | 47.83 |
